# Nuclear pore complex dysfunction drives TDP-43 pathology in ALS

**DOI:** 10.1016/j.redox.2025.103824

**Published:** 2025-08-14

**Authors:** O. Ramírez-Núñez, S. Rico-Ríos, P. Torres, V. Ayala, A. Fernàndez-Bernal, M. Ceron-Codorniu, P. Andrés-Benito, A. Vinyals, S. Maqsood, I. Ferrer, R. Pamplona, M. Portero-Otin

**Affiliations:** a Metabolic Pathophysiology Research Group, Dept of Experimental Medicine, University of Lleida-IRBLleida, Avda Rovira Roure, 80 E25196, Lleida, Spain; b Cognition and Behaviour Research Group, IRBLleida, Avda Rovira Roure, 80, Lleida, E-25196, Spain; c Network Centre of Biomedical Research of Neurodegenerative Diseases (CIBERNED), Institute of Health Carlos III, L'Hospitalet de Llobregat, 08907, Barcelona, Spain; d Neurology and Neurogenetics Group - Neuroscience Program, Bellvitge Biomedical Research Institute (IDIBELL), L'Hospitalet de Llobregat, 08907, Barcelona, Spain; e Department of Pathology and Experimental Therapeutics, University of Barcelona, Gran Via de l'Hospitalet, 199 L'Hospitalet de Llobregat, Barcelona, 08908, Spain; f Reial Academia de Medicina de Catalunya, Barcelona, Spain

## Abstract

Amyotrophic lateral sclerosis (ALS) is a fatal neurodegenerative disease characterized by progressive motor neuron degeneration and pathological aggregation of TDP-43. While protein misfolding and impaired autophagy are established features, accumulating evidence highlights the nuclear pore complex (NPC)as a vulnerable, redox-sensitive hub in ALS pathogenesis. Here, we show that selective loss of NPC components, particularly the scaffold proteins NUP107 and NUP93, and FG-repeat-containing components—is a consistent finding across ALS postmortem spinal cord, SOD1^G93A and TDP-43 mutant mouse models, and human cell systems.CRISPR-mediated depletion of NUP107 in human cells triggers hallmark features of ALS pathology, including cytoplasmic TDP-43 mislocalization, increased phosphorylation, and autophagy dysfunction. Conversely, TDP-43 knockdown perturbs NPC composition, suggesting a reciprocal regulatory loop. Crucially, we demonstrate that oxidative stress exacerbated NPC subunit mislocalization and enhanced TDP-43 aggregation. Using oxime blotting and DNPH assays, we show that FG-repeat subunits of NPC were direct targets of redox-driven carbonylation, indicating that oxidative modifications compromise NPC integrity thuspotentially affecting nucleocytoplasmic transport. Our findings established NPC dysfunction as a redox-sensitive driver of TDP-43 pathology in ALS and highlight nucleocytoplasmic transport as a promising therapeutic axis. The susceptibility of long-lived NPC proteins to oxidative damage provides a mechanistic link between redox stress, proteostasis collapse, and neurodegeneration.

## 1 Introduction

Amyotrophic Lateral Sclerosis (ALS) is a devastating neurodegenerative disorder characterized by the progressive degeneration of motor neurons in the brain and spinal cord, leading to severe muscle weakness, disability, and ultimately respiratory failure [1]. Despite extensive research, the etiology of ALS remains poorly understood, with both genetic and environmental factors implicated in its onset and progression. Recent studies have illuminated the role of nucleoporins (NUPs) —key components of the nuclear pore complex (NPC)—as significant contributors to the underlying cellular mechanisms of ALS[[2], [3], [4], [5]]. NPC is essential for regulating nucleocytoplasmic transport, a critical process for maintaining cellular homeostasis and function: depending on the size of the cargo, nucleocytoplasmic transport occurs through NPC, embedded in the double nuclear membrane [6,7]. Each NPC is composed of multiple copies of approximately 30 different NUPs arranged in octagonal rotational symmetry around the central transport channel [8], and it's a large complex, varying from 60 MDa in yeast and up to 110 MDa in mammals [9].

Among the NPC components, several subcomplexes arise as important, such as the NUP107-NUP160 and the NUP93-NUP205, which contain the intracellular proteins of longer life [10]. The ring composed of NUP107-NUP160 is the largest protein complex in size and complexity and is required in the first assembly steps of the NPC [11], though the assembly itself of these complexes is a poorly characterized process [12]. Attached to the inner side of these structures, in the central transport channel, there are proteins characterized by containing native phenylalanine-glycine domains, termed FG-repeat containing NUPs. These proteins establish a selective permeability barrier that regulates transport through the NPC by interacting these domains with proteins involved in nucleocytoplasmic transport [13].

Disruptions in NPC structure have been linked to motor neuron degeneration, with specific mutations in NUPs correlating with increased neuronal death and altered RNA metabolism [14]. Dysfunction of NPC may exacerbate ALS pathology by impairing RNA transport and metabolism, particularly through their interactions with TDP-43, a hallmark of ALS when aggregating in the cytoplasm [3,15]. Indeed, loss of homeostatic RNA metabolism, potentially related to NPC alteration, is a common mechanism in the ALS/frontotemporal dementia (FTD) continuum, resulting in the mislocalization and aggregation of specific proteins related to RNA biology (reviewed in Ref. [16]). One could count NPC subunits among these aggregated proteins, closing a vicious circle. For instance, cytoplasmic NUPs, coat NUPs, inner ring NUPs, central channel NUPs, and basket NUPs were all found to co-aggregate with pathological TDP-43 C-terminal fragments [17]. Also, except for POM121, each NUP co-aggregating with TDP-43 contains FG-domains. Of note, mutations in components of NPC leading to ALS suggest that disruptions in nucleocytoplasmic transport mediated by NUP loss may contribute to motor neuron degeneration. For instance, variants in the NUP50 gene have been linked to decreased protein levels and increased neuronal death [18]. Also, mutations in the *GLE1*, encoding an NPC-located protein involved in RNA processing, are associated with ALS. At least two mutations in this gene have been identified, and both caused a decrease in the GLE1 protein in the NPC [19].

Besides genetic background, aging is considered the main risk factor for ALS. Interestingly, the efficiency and selectivity of transport nucleocytoplasmic deteriorate significantly during aging [20,21]. This is because several NUPs, such as members of the NUP107-NUP160 complex, are among the longest-lived proteins in post-mitotic neurons [22]. Post-mitotic cells cannot repair them, and damaged NPC accumulate during cell life, causing damage to the structure of the core and alteration of transport nucleocytoplasmic. In addition, oxidative stress, related to aging and involved in various neurodegenerative disorders [23], could damage to NPC components. Of note, oxidative stress has been implicated in multiple neurodegenerative mechanisms, including those mediated by the YAP-TFR/ROS pathway, as shown in renal and neuronal systems [24].

Here, we present evidence that ALS is associated with a significant decrease in specific NUP content in the spinal cord and brain cortex, particularly in FG-rich NUPS and NUP107, as demonstrated by Western blot, confocal microscopy, and RNA sequencing. These findings support the hypothesis that NPC dysfunction could be an integral component of ALS pathology, contributing to TDP-43 aggregation and altering cellular stress responses, including autophagy. Understanding this interplay could provide novel therapeutic avenues targeting nuclear transport mechanisms in ALS and related neurodegenerative diseases.

## 2 Materials and methods

### 2.1 Human samples

Post-mortem spinal cord tissue was obtained from patients diagnosed with ALS, as well as from control subjects without a clinical or pathological history of neurological disease. These samples were provided by the Neurological Tissue Bank from the Neuropathology Institute- (Hospital de Bellvitge, Universitat de Barcelona). Tissue samples were preserved at −80 °C for subsequent processing. All patients and/or their next of kin provided informed consent for tissue donation. Additionally, demographic and clinical data, including sex, age at diagnosis, age at time of death, and cause of death, were collected (Table 1 and Supplemental Table 1). In all cases, diagnosis was confirmed through extensive neuropathological studies in accordance with established criteria [25]. Samples with extended post-mortem intervals or potential tissue degradation were excluded. For immunohistochemistry, post-mortem samples were obtained from the Institute of Neuropathology HUB-ICO-IDIBELL Biobank (C.0008091) following the guidelines of Spanish legislation on this matter and the approval of the CEIC of the Bellvitge University Hospital (Ref.PR010/22). The post-mortem interval between death and tissue processing was between 2 h and 17 h and not belong to CSF patient's cohort. Tissue processing was carried out as detailed elsewhere [26]. All cases met the neuropathological criteria for classical ALS. Patients with any associated pathology were excluded. Age-matched cases, who had not suffered from neurologic or psychiatric diseases and did not have abnormalities in the neuropathological examination, were also assessed as controls. Single-label immunohistochemistry was performed on de-waxed sections of spinal cord of ALS (n = 3) and control cases (n = 3) that were 4-μm-thick. The sections were subjected to boiling in citrate buffer (pH = 6.0) for 20 min to enhance antigenicity. Subsequently, endogenous peroxidases were blocked by treating the sections with Dako Real peroxidase-blocking solution for 15 min at room temperature. Next, Dako Real diluent solution (Dako) was applied to block non-specific binding, permeabilize the tissue, and dilute the primary antibodies TRP and NUP107, according manufacturer instructions. Thesections were incubated with the primary antibodies overnight at 4 °C. Following incubation, the slides were washed and then incubated with a biotinylated secondary antibody “Ready to use” (Abcam), for 30 min at room temperature. This was followed by incubation with Streptavidin Peroxidase solution “Ready to use”(Abcam), for an additional 30 min at room temperature. The peroxidase reaction was visualized using diaminobenzidine and H2O2, resulting in a brown precipitate indicating immunoreactivity. The sections were counterstained with hematoxylin. To ensure the specificity of the immunostaining, a control was performed by omitting the primary antibody, which resulted in no signal upon incubation with only the secondary antibodies.

**Table 1:** Demographics of human samples.

Sex	Age	Diagnosis	ALS type	Disease duration (years)	Post-mortem delay (hours:min)
**M**	64	ALS	Bulbar	2	16:30
**M**	57	ALS	Bulbar	2	4
**F**	75	ALS	Bulbar	4	4:50
**F**	79	ALS	Bulbar	1.5	2:10
**F**	57	ALS	Spinal	5	10
**M**	50	ALS	Spinal	5	10:10
**M**	75	ALS	Bulbar	2	3
**M**	71	ALS	Spinal	8	3:25
**M**	68	Control	–	–	10:55
**M**	64	Control	–	–	08:35
**M**	59	Control	–	–	11:20
**F**	59	Control	–	–	11:20
**F**	79	Control	–	–	
**F**	66	Control	–	–	4:55h
**F**	76	Control	–	–	06:30
**F**	75	Control	–	–	06:10

### 2.2 Animal models

All animal studies complied with institutional and national guidelines for the care and use of laboratory animals, procedure number 9455, supervised by the local government Generalitat de Catalunya. Three different transgenic mice models of ALS were obtained from the Jackson Laboratories (Bar Harbor, ME, USA) along with littermate non-transgenic controls, including one carrying the human *SOD1*^G93A^ mutation (JAX002726, from now on G93A mice), one with the human *TARDBP*^Q331K^ mutation associated to the Prp promoter (JAX017933, Q331K) and one expressing a truncated form of TDP-43 with a neurofilament heavy promoter under doxycycline induction (JAX028413,NLS-TDP43) were used, In the case of G93A and Q331K, both sexes of mice were included. Animals were housed in temperature- and humidity-controlled conditions on a 12-h light/dark cycle and given ad libitum access to food and water. In the case of the NLS-TDP43 strain, mice were fed with chow containing doxycycline (200 mg/kg) (S3888, Bio-Serv,Flemington, NJ, USA) to avoid transgene expression. At weaning (20–22 days of age), mice were switched to regular chow (S4207, Bio-Serv), not containing doxycycline (dox-off) to allow the expression of the transgen. After euthanizing mice, spinal cord samples were obtained according to authorized procedures at specified at presymptomatic (60 days), symptomatic (100 days), and late-stage disease (130 days) time points (for G93A); at 150 days (presymptomatic) for the Q331K mice, and at around 18–21 days (loss of hindlimb reflex for the presence of both hindlimbs held together within 5 s of being raised) after dox-off, for the NLS-TDP43 mice.

### 2.3 Cell culture

Experiments were performed with both murine and human cell lines. Mouse neuroblastoma Neuro-2A cells (CCL-131, ATCC, Manassas, VA, USA) were maintained according the provider advice. hIPSC control cell line, obtained from NINDS Human Genetics DNA and Cell line Repository (ND41865, Coriell Institute, Camden, NJ, USA), HeLa (CCL-2, ATCC) and HEK-293 (CRL-1573, ATCC) cells were cultured under similar conditions or according to the supplier's recommendations. Unless stated otherwise we maintained cells in DMEM (10564011, Thermo Fisher Scientific, Waltham, MA, US)(or a 1:1 mixture of DMEM/F12, 11320033, Thermo) supplemented with 10 % fetal bovine serum (A5256701, Thermo), 100 U/mL penicillin, and 100 μg/mL streptomycin (15070063, Thermo). Cells were routinely tested for mycoplasma contamination, that was prevented employing Plasmocin® prophylactic in culture media(ant-mpp, Invivogen, San Diego, CA).

We developed a HeLa cell line expressing an inducible system to knockdown *TARDBP* employing the Tet-pLKO-puro plasmid (Plasmid #21915, Addgene, Watertown, MA, USA) [26], as described [27]. These cells, upon doxycycline exposure, express a short hairpin RNA (shRNA) targeting TARDBP mRNA (Millipore-Sigma TRCN0000016038). Lentiviral particles were produced in HEK293T cell lines transfected with psPAX2 (Addgene, 12260) and pMD2.G (Addgene, 12259) plasmids (both a gift from Dr. Trono) and Tet-pLKO-puro-TARDBP for 3 days. Cell medium containing lentiviral particles was collected, centrifuged at 500 relative centrifugal force for 5 min, and filtered through a 0.45 μm pore size membrane. HeLa cells were transduced and selected with puromycin (P9620, Sigma) at 1 μg/ml. A single clone was isolated to obtain the HeLa pLKO cell line.All cultures were kept at 37 °C in a 5 % CO_2_ atmosphere.

To investigate the role of specific NUPs, CRISPR/Cas9 constructs targeting NUP107 were designed using guide RNAs against human NUP107 gene (Santa Cruz Biotechnology, Dallas, TX, US, Cat #sc-405252-KO-2). Cells were seeded to reach 50–70 % confluence at transfection. Lipid-based transfection reagents (Lipofectamine®3000, Thermo) were used according to the manufacturer's protocol. Puromycin (700 μg/mL) was employed for selecting a stably transfected clone. For transient transfection we employed RNAiMAX (13778100, Thermo Fisher Scientific) following manufacturer instructions and a siRNA targeting NUP107 (siNUP107) at 20 nM with the following sense strand sequence GCUGCAAAAGAAGUAUUUG previously described in Ref. [27] or a scrambled siRNA an 20 nM [28] UAAGGCUAUGAAGAGAUAC. Cells were silenced for 48 h. Successfully edited clones or transiently transfected pools were evaluated by qRT-PCR and Western blot to confirm NUP107 knockdown.

To induce the different types of cell stress associated to TDP-43 aggregation, we employed the conditions indicated in Table 2. In each case, vehicle-treated cells served as controls. For nutrient starvation, cells were treated with HBSS medium for 4 h. To evaluate the interaction between NPC functions and TDP-43, we employed Leptomycin B (L2913, Sigma) -an inhibitor of nuclear export-, and Importazole (SML0341, Sigma), specifically blocking importin-β-mediated nuclear import.

**Table 2:** Treatments for cell stress.

Stress Type	Reagent	Concentration^a^	Time	Cell
Oxidative stress	Paraquat	1 mM	24 h	SH-SY5Y
	Menadione			
	Sodium Arsenite	0.5 mM	1h	SH-SY5Y
	H_2_O_2_	10 μM	4h	Neuro 2a
Osmotic stress	Sorbitol	400 mM	3 h	HEK-293/IPSc
Endoplasmic reticulum stress	Thapsigargin	5 μM	4 h	Neuro 2a
Proteasome stress	MG132	10 μM	4 h	Neuro 2a

a Oxidative stress and other stress conditions (e.g., 10 μM H_2_O_2_) were selected based on prior studies reporting redox imbalance and NPC injury in neuronal and non-neuronal cells. While effective for inducing measurable effects, we did not perform dose–response or time-course analyses for all stressors, which we acknowledge as a limitation.

For the assessment of cell viability, we employed the PrestoBlue™ Cell Viability Reagent (Invitrogen, A13262), based on the reducing power of living cells. PrestoBlue™ reagent was added at a ratio of 1:10, and cells were incubated for 1h at 37 °C in a humidified environment with 5 % CO2. After incubation, samples were read using a fluorescence microplate reader (Infinite M200, TECAN) at 560/590 nm excitation/emission wavelengths.

### 2.4 Isolation of nuclei from tissue

Cell nuclei were extracted from portions of the spinal cord or frontal cortex, following modifications of the protocol by Blobel and Potter [29], as recently explained [28]:. Briefly, approximately 0.24 g of frozen tissue was used, which was allowed to thaw at 4 °C. Once thawed, the tissue was cut into very small pieces using a scalpel and transferred to a 2 mL Eppendorf tube, where it was homogenized in two volumes of cold 0.25 M sucrose in TKM buffer (0.05 M Tris, 0.025 M KCl, 0.005 M MgCl2) and commercial protease and phosphatase inhibitors (Thermo, 78440). Homogenization was initially performed using a mechanical Polytron homogenizer with a plastic plunger, applying approximately 20–40 strokes. Subsequently, the homogenate was transferred to a Dounce homogenizer with a glass plunger, and 20–40 strokes were performed using the tight (B) plunger. After this step, the homogenate was transferred to an ultracentrifuge tube (Beckman Coulter, Brea, CA, USA 344057), and 1080 μL of 2.3 M sucrose in TKM buffer was added. The contents of the tube were mixed 4–5 times by inversion. A pipette was then carefully inserted into the bottom of the centrifuge tube, and 540 μL of 2.3 M sucrose in TKM buffer was gently under layered, ensuring the formation of two well-defined sucrose phases, with the homogenate remaining in the upper phase. The ultracentrifuge tubes were balanced to avoid imbalance during centrifugation and were centrifuged at 124,000 g at 4 °C f or 30 min in an Optima L-100XP ultracentrifuge (Beckman Coulter) with an SW 55 Ti rotor. The resulting pellet, which is barely visible, was resuspended in 50 μL of TKM buffer containing commercial protease and phosphatase inhibitors (Thermo Scientific 78440). The presence of nuclei was confirmed by fluorescence microscopy after staining with DAPI at a concentration of 1 μg/mL and by Western blot as indicated previously [28,28].

### 2.5 Immunofluorescence

For microscopy, approximately 50,000–80,000 cells were seeded onto coverslips previously treated with 1 mg/mL Poly-l-Lysine (Sigma, P1274). After indicated treatments, cells were washed 3 times with PBS and fixed with 4 % paraformaldehyde (PFA) dissolved in PBS for 15 min at room temperature. After fixation, the cells were incubated with a permeabilization and blocking solution (0.2 % Triton X-100 (v/v), 2 % BSA (v/v), and 2 % horse serum (v/v) in PBS). Subsequently, the cells were incubated overnight at 4 °C with the appropriate primary antibodies, diluted in 1/10 blocking solution in PBS, in a humid chamber.After this incubation period, the cells were washed three times with 1x PBS and incubated with the appropriate secondary antibody and 1 μg/mL DAPI (Sigma D9542) for nuclear staining, for 1 h at 4 °C. Finally, the cells were washed three times for 5 min each with PBS and mounted onto slides using Fluoromount-G mounting medium (0100-01, Southern Biotech, Birmingham, AL, USA) and left to dry at room temperature.

### 2.6 Immunofluorescence of isolated nuclei

The isolated nuclei were fixed in 4 % (w/v) PFA in PBS at a ratio of 1/3 (nuclei suspension/4 % PFA solution (v/v)) for 15 min. Subsequently, the mixture was pipetted onto a round coverslip with a diameter of 12 mm, which had been previously placed in 2 cm^2^ culture plates. The plate containing the nuclei was centrifuged in a centrifuge suitable for culturing plates at 1000 rpm for 3 min. After centrifugation, the coverslips were carefully removed using fine tweezers and placed on a parafilm-covered support. The nuclei were incubated in a permeabilization and blocking solution (0.1 % Triton X-100, 10 % normal goat serum in PBS) for 30 min. Following this, three gentle washes with PBS were performed, taking care not to dislodge the nuclei. The coverslips/nuclei were then incubated overnight at 4 °C with the appropriate primary antibodies, diluted in 1/10 blocking solution in PBS, in a humid chamber. After this incubation period, the samples were washed three times with PBS and incubated with the appropriate secondary antibody diluted in PBS and 1 μg/mL DAPI for nuclear staining, for 1 h at 4 °C. Finally, three 5-min washes with PBS were performed, and the nuclei were mounted onto slides using Fluoromount-G mounting medium and left to dry at room temperature.

### 2.7 Quantitative analyses of microscopy images

To obtain images of the cellular and nuclear staining, an Olympus fluorescence microscope equipped with epifluorescence and a DP70 CCD camera, and a Olympus FV1000 confocal microscope, were employed. All conditions to be compared (i.e. vehicle vs stressor) were acquired employed the same microscope settings. Images were acquired for fluorescence quantification and analyzed using the open-source software CellProfiler version 2.1.1 for Windows [30], performed in a double blinded fashion.

### 2.8 Western-blot

For cell culture experiments, after treatment, cells were washed three times with cold PBS to remove residual proteins and traces of culture medium. Subsequently, 100–300 μL of RIPA lysis buffer, supplemented with commercial protease and phosphatase inhibitors (Thermo Scientific 78440), was added to the plate. The cells were scraped off using a cell culture scraper, and the lysate was transferred to an Eppendorf tube. The lysate was sonicated for 1 min to disrupt cellular structures. Finally, the samples were stored at −20 °C until protein analysis.

Protein extraction from tissues was performed using mechanical homogenization with an Ultra-Turrax homogenizer (L_IKA_3001, IKA-Werke GmbH & Co. KG, Staufen, Germany). Approximately 50 mg of tissue was placed in a conical-bottom Eppendorf tube and kept on ice. Then, 500 μL of RIPA buffer (containing freshly added protease and phosphatase inhibitors) was added, and the tissue was homogenized using the homogenizer for approximately 20 s, depending on the tissue's hardness. The homogenate was sonicated for 30 s to disrupt tissue structures. The samples were stored at -20 °C until protein analysis.

In all cases protein concentration was determined using the Bradford assay (Bio-Rad, Hercules, CA, USA, Protein Assay Kit I, 5000001). Samples were then mixed with sodium dodecyl sulfate (SDS) and beta-mercaptoethanol, heated at 95 °C for 5 min, and 15 μg of protein per sample was loaded onto SurePAGE™ Precast gels (4–20 %, 15 wells, GenScript, Piscataway, NJ, USA) for Sodium Dodecyl Sulfate Polyacrylamide Gel Electrophoresis (SDS-PAGE). Following electrophoresis, proteins were transferred to polyvinylidene difluoride membranes using an EZ-Transfer device (GenScript). The membranes were blocked with I-Block (Thermo, T2015) for 1 h, followed by incubation with primary antibodies overnight at 4 °C and secondary antibodies for 1 h at room temperature. Immunoreactive bands were detected using chemiluminescence with Immobilon™ ECL Ultra Western HRP Substrate (Merck Millipore, WBKLS0500) and visualized using a Chemidoc MP Imaging System (Bio-Rad).

For normalization, membranes were stained with Coomassie Brilliant Blue G (Sigma-Aldrich, 27815), or No-Stain™ Protein Labeling Reagent (before blocking) and specific protein bands were quantified using ImageLab software (Bio-Rad, version 6.1).

### 2.9 Immunoprecipitation

For co-immunoprecipitation of endogenous FG-NUPs patients frontal cortex lysed in oxime blot RIPA lysis buffer (see below), sonicated at 4 °C for 30 min in an ultrasound bath, and the lysate precleared with 25 μL of protein G dynabeads™. FG-NUPs were immunoprecipitated with mouse monoclonal antibody mab414 conjugated to 50 μL of cleared protein G dynabeads™ (Invitrogen) for 10 min at RT. An anti-IgG mouse monoclonal antibody (Thermo) was used as a negative control. Beads were washed twice with buffer before the addition of 200 μg of sample protein and incubated at 4 °C overnight, washed three times with buffer, and resuspended with appropriate buffer for the downstream method.

### 2.10 Oximeblot, DNPH and prediction of carbonylated residues

The oxime blot method, for detection of oxidatively modified proteins, was performed following the protocol described previously [31]. Briefly, proteins in the cell lysate (HEK293 or HeLa) were extracted using oximeblot RIPA buffer, and 8 μL of the sample (1.25 μg/μL), 1 μL of biotin-aminooxy (100 mM in DMSO), and 1 μL of pPDA (0.5 M in DMSO with 5 mM DTT) were incubated at room temperature (25 °C) at 500 rpm for 3 h in a Thermomixer (Eppendorf, Hamburg, Germany). To stop the reaction, 1 μL of hydroxylamine (1 M in DMSO) was added, and the sample was prepared with loading buffer (2x), heated at 95 °C for 5 min, and stored at −20 °C until use. For immunoprecipitated samples from brain cortex homogenates, the beads (the presented data is for two gels) were resuspended in 16 μL of phosphate buffer (pH 7.0) and incubated with 2 μL of biotin-aminooxy (100 mM in DMSO) and 2 μL of pPDA (0.5 M in DMSO with 5 mM DTT) at room temperature (25 °C) at 500 rpm for 3 h in a Thermomixer (Eppendorf). To stop the reaction, 2 μL of hydroxylamine (1 M in DMSO) was added, and the sample was prepared with loading buffer (2x), heated at 70 °C for 10 min, and stored at −20 °C until use.

The DNPH method followed the protocol described in Ref. [32], with minor modifications to adapt it to immunoprecipitated samples. Briefly, proteins were extracted using RIPA lysis buffer, and 200 μg of protein was immunoprecipitated in a volume of 100 μL as previously described. For the positive oxidation control, 5 μL of H_2_O_2_ (37 %) was added and incubated at RT for 5 min. The sample was eluted from the magnetic beads using 20 μL of 6 % SDS at 70°C for 10 min. A spin was performed, and the sample was left to rest at RT for 10 min. DNP solution was added for exactly 7 min at RT. The reaction was stopped by adding 25 μL of RS solution, followed by Western blot analysis. For the prediction of potentially carbonylated residues, we employed two strategies. First we manually searched for NUPs of interest, for TPR and for TFEB in the CarbonylDB for experimentally validated carbonyl modifications in proteins [33]. We also searched the nucleoporin, TPR and TFEB sequences in the iCarsPS webserver [34].

### 2.11 RNA extraction and transcriptome analysis using RNA-seq

Frozen samples of the anterior horn of the lumbar spinal cord (n = 9 sALS and n = 6 controls) were obtained for RNA extraction using RNeasy Mini Kit following the instructions of the supplier (Qiagen® GmbH, Hilden, Germany). RNA integrity and 28S/18S ratios were determined with the Agilent Bioanalyzer (Agilent Technologies Inc, Santa Clara, CA, USA) to assess RNA quality, and the RNA concentration was evaluated using a NanoDrop™ Spectrophotometer (Thermo Fisher Scientific, Barcelona, Spain). The transcriptomic analysis was carried out using RNA-seq technology. Following the manufacturer's protocol, RNA libraries were prepared using the NEBNext® Ultra II RNA Library Prep Kit (New England Biolabs). A total of 15 samples of RNA were validated with an RNA High Sensitivity Tape Station (Agilent Technologies). A quantity of 700 ng of RNA was used to prepare the libraries, which were validated using a DNA 1000 Tape Station Kit. Equimolar proportions of each library were mixed, and the subsequent pool was quantified by qPCR and sequenced in 2 NextSeq High Output 2 × 75 runs (Illumina). Counts were obtained with HTSeq software (version 2.0.2). Reads were mapped using STAR software against the human GRCh38, which was obtained from ENSEMBL release 99, and the corresponding GENCODE annotation v33. BAM files from all runs in each sample were merged using MergeSamFiles, and duplicated reads were marked and removed using MarkDuplicates from Picard tools v2.18.12. Quality control was performed using FASTQC while studying running variables and group cofactors. The protein-coding genes were selected for further analysis. At the expression level, no deviation was observed. A filter of lowly expressed genes by the mean values of each gene's expression (log2CPM >1) was additionally performed. Genes with low expression were filtered out for further analysis. Finally, we fitted a linear regression model between groups to obtain differentially regulated genes. Then, the p-values for the coefficient of interest were adjusted for multiple testing. This method, which controls the expected false discovery rate (FDR) below the specified value, is the default adjustment method because it is the most likely to be appropriate for microarray studies, finally obtaining the significant adjusted p-values (p < 0.05) for each gene.

### 2.12 Quantitative reverse transcription PCR (RT-qPCR)

To quantify TDP-43 splicing dysfunction, based on the accrual of cryptic exons in selected mRNAs [33], total RNA was isolated from cells with TRI Reagent (Thermo Fisher Scientific, AM9738) following the manufacturer's protocol. The RNA concentration was then determined on a NanoDrop ND-1000 (Thermo Fisher Scientific). One microgram of RNA was then reverse-transcribed using TaqMan Reverse Transcription Reagent (Thermo Fisher Scientific, N8080234) and random hexamers. RT-qPCR assays were conducted on a CFX96 instrument (Bio-Rad) using SYBR Select Master Mix for CFX (Thermo Fisher Scientific, 4472937). Each 20 μL reaction consisted of 4 μL cDNA, 10 μL SYBR Select Master Mix, 0.2 nM forward primer, 0.2 nM reverse primer, and 4 μL PCR-grade water. The thermal program included a 2-min step at 50 °C, followed by 2 min at 95 °C, then 40 cycles of 95 °C for 15 s and 60 °C for 1 min. A melting curve analysis was subsequently performed from 65 to 95 °C at increments of 0.1 °C per second. Primers used in these experiments are listed in Supplemental Table 2. Cryptic exon inclusion, or Percentage Spliced-In (PSI), was calculated using the formula: 100 × 2^(−[Conserved exon Cq − Cryptic exon Cq]).

### 2.13 Flow cytometry

Flow cytometry was used to analyze nuclear proteins in isolated nuclei according to the following protocol. For this purpose, the antibodies NUP107, NUP93, and MAB414 were conjugated with the fluorophores R-Phycoerythrin (RPE), allophycocyanin (APC), and APC/Cy7 using the Abcam conjugation kits ab102918, ab201807, and ab102859, respectively, following the manufacturer's recommendations and instructions. In general, 1 μL of fluorophore modification reagent (supplied in the conjugation kit) was added to 10 μL of the antibody to be modified and gently mixed. The cap of the conjugation mixture vial (supplied in the conjugation kit) was removed, and the antibody sample (with the added RPE,APC or APC/Cy7 modification reagent) was pipetted directly onto the lyophilized material (supplied in the conjugation kit). The liquid was then resuspended once or twice using a pipette and left to incubate for 3 h in the dark. Afterward, 1 μL of Quencher reagent (supplied in the conjugation kit) was added for every 10 μL of antibody used and gently mixed. The conjugates were used after 30 min, as indicated by the conjugation kit. Briefly, a nuclei solution in PBS was prepared at a ratio of 1:20 (nuclei suspension:PBS (vol:vol)). The fluorophore-conjugated antibody was then added at a 1:500 dilution and incubated for 1 h at room temperature in the dark. Subsequently, the nuclei were analyzed using the FACS-Canto II digital cytometer (BD Biosciences).

### 2.14 Statistical analysis

All experiments were performed in at least three independent biological replicates, unless stated otherwise. Number of replicates are present in figures. Data were tested for normality (Shapiro–Wilk or Kolmogorov–Smirnov) and homoscedasticity. Parametric or nonparametric tests (Student's *t*-test, one-way or two-way ANOVA with post hoc comparisons, Mann–Whitney, or Kruskal–Wallis) were used as appropriate. GraphPad Prism (v 10.4.1) or SPSS (v 27) was employed for analyses. P-values <0.05 were considered significant. Blinding for image experiments was unreleased after statistical analysis.

## 3 Results

### 3.1 ALS is associated to altered nucleoporin content in spinal cord and brain cortex

**Fig. 1:**
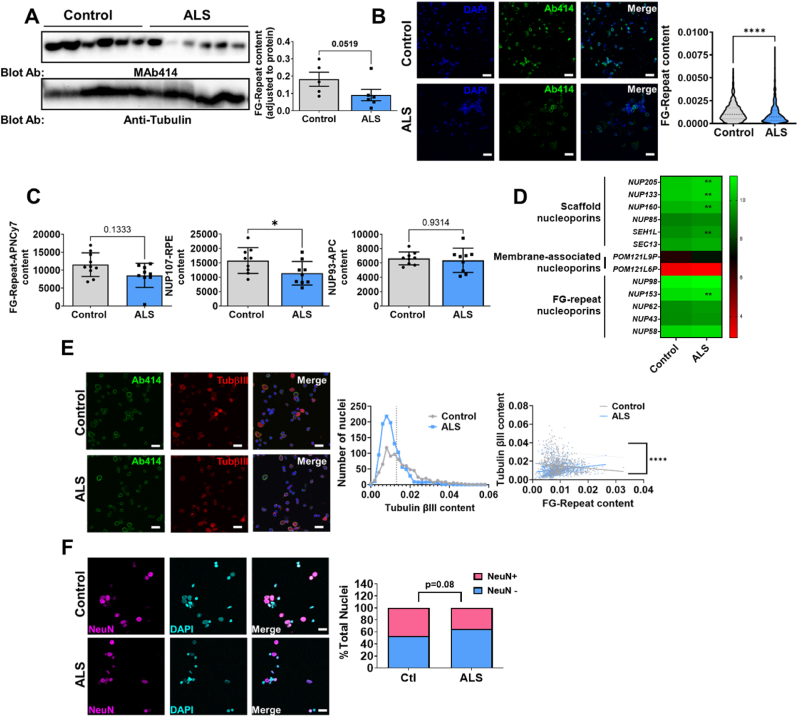
**ALS is associated to altered nucleoporin content in spinal cord and brain cortex**. A) Left panel shows a representative western-blot image of FG-repeats containing NUPs (reactive to monoclonal antibody MAb414) of lumbar spinal cord homogenates from ALS patients and healthy, age and sex matched controls. Right panel shows the quantified immunoreactivity. B) The left panel shows representative confocal microscopy images from isolated nuclei from spinal cord samples, quantified in the violin plots from the right panel, demonstrating *in situ* decreased nucleoporin content.C) Quantitative analyses of NUP107, FG-repeats, and NUP93 in isolated nuclei by flow cytometry analyses, showing decreased content of NUP107 in spinal cord samples from ALS patients. D) RNA Seq data in heatmap showing that the mRNAs content of scaffold NUPs and NUP153 (an FG-repeat containing NUP) was decreased in spinal cord from ALS patients.Right panel indicates TPM (transcrits per kilobase million). E) Left panel shows confocal microscopy images with anti-tubulin β III immunoreactivity in nuclei isolated from brain cortex (area 8) from ALS patients and healthy individuals. Middle panel indicates tubulin β III content distribution, with the vertical line situating median values. Right panel illustrates differences in relationship between the content of FG-repeat NPC and tubulin β III in nuclei, with reference to ALS and tubulin β III content. F) Left panel shows representative confocal microscopy images from isolated nuclei from spinal cord samples, quantified plots from the right panel, demonstrating a decreased amount of nuclei from neuronal cells, based on NeuN content. Bars show mean values ± SEM or % in F. ∗,∗∗, and ∗∗∗∗ indicate p < 0.05, p < 0.01 and p < 0.0001 with reference to control values by Student's T test, Chi-Square (F) or Mann-Whitney *U* test. In E) For controls, linear relationship equation Y = −0.2721∗X + 0.01782 (p < 0.0004) and for ALS, the equation is Y = 0.3195∗X + 0.008008 (p < 0.0001). Scale white bar length in B, E and F are 50 μm long. Ns: non statistically significant differences.

**Fig. 2:**
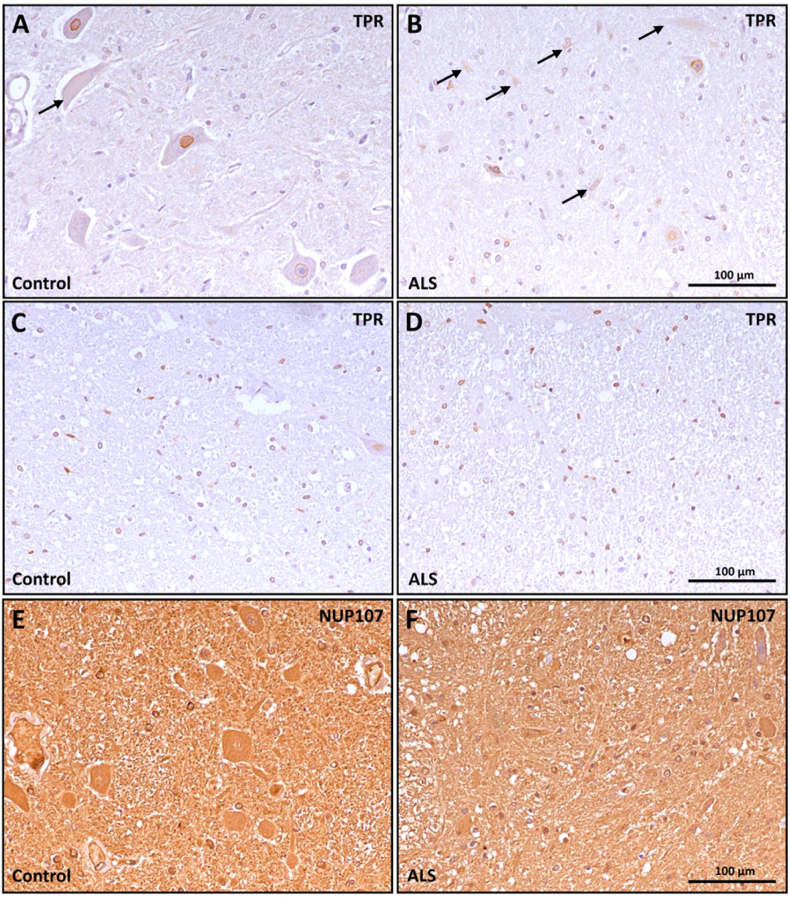
**NUPs are present in motor neurons and glial cells in human lumbar spinal cord, ventral horn.** Representative immunohistochemical images of cellular distribution of the scaffolding NUP TPR (A–D) and the FG-repeat NUP107 (E,F). Samples from grey matter are shown in A,B,E and F, while as white matter cells (i.e. glial cells) are shown in C and D. Samples from healthy donors (A,C and E) and ALS patients (B,D and F) are presented. Arrows in A and B indicate non-nuclear staining of TPR, which were more frequent in ALS samples.

To determine if ALS is accompanied by changes in NPC that can contribute to alterations in nucleocytosolic transport, Western blot and immunocytochemical analysis were performed in samples from ALS. FG-repeats containing NPC were quantified using the monoclonal antibody Mab414, showing its decrease in ALS spinal cord samples (Fig. 1A). Likewise, isolated nuclei extracted from the spinal cord (Fig. 1B) showed a significant decrease in the immunoreactivity to Mab414, suggesting that nuclear envelopes from ALS samples have decreased FG-repeat content. To further characterize this phenomenon employing an independent assay of other NPC, we set up a flow cytometry method for evaluating NUP107, NUP93, and FG-repeats in isolated nuclei. The results (Fig. 1C) suggested a tendency towards decreased concentration of FG-repeats (p = 0.13) and significantly decreased content of NUP107 in ALS. These results were not explained by decreased expression of mRNAs belonging to NPC, as we could not determine NUP107 mRNA by RNA-Seq (Fig. 1D). Interestingly, the mRNA of the scaffold NUPS showed significantly decreased values in ALS samples, while the other components (membrane-associated NUPs and FG-repeat containing NUPs) did not show major differences (Fig. 1D). To evaluate whether this was tissue-specific, we studied the motor cortex samples from the same patients. In these analyses, considering that neuronal nuclei with damaged envelopes allow the entry of cytosolic tubulin βIII [20], we quantified the amount of tubulin βIII positive nuclei (i.e. with damaged nuclear envelope), and we found that ALS was associated a lower number of nuclei with a high tubulin βIII (Fig. 1E). This was also linked to changes in the relationship between FG-repeats NPC and tubulin βIII content in those ALS nuclei (Fig. 1E). Thus, in control conditions we observed an expected negative correlation between tubulin βIII in nuclei and FG-repeats NPC, while as for ALS, this relationship was lost (Fig. 1E). Indeed, in the interpretation of tubulin βIII in nuclei, we should take into account that tissue samples from ALS patients had globally lower number of nuclei from neuronal cells (Fig. 1F). To ascertain these changes *in situ*, we performed immunohistochemistry analyses of NUP107 and TPR, a scaffolding NUP in spinal cord samples from ALS patients. The results (Fig. 2) demonstrate that TPR NUP is highly expressed in spinal cord motor neurons in control conditions, without evident changes in ALS patients, neither at grey matter (Fig. 2A and B) nor at the white matter of anterior horn of spinal cord, where nuclei (i.e. glial cells) showed also high staining of TPR (Fig. 2C and D). Of note, some remaining motor neurons in ALS samples showed some cytosolic TPR distribution (Fig. 2B). Regarding NUP107, we observed decreased immunostaining, coincident with lower motor neuron count in ALS samples (Fig. 2E and F). Globally, these results suggest that ALS is associated with the loss of NPC components, particularly NUP107 and NPC-containing FG-repeats.

### 3.2 SOD and TDP-43-based ALS preclinical models show sex-dependent altered nucleoporin content in spinal cord

**Fig. 3:**
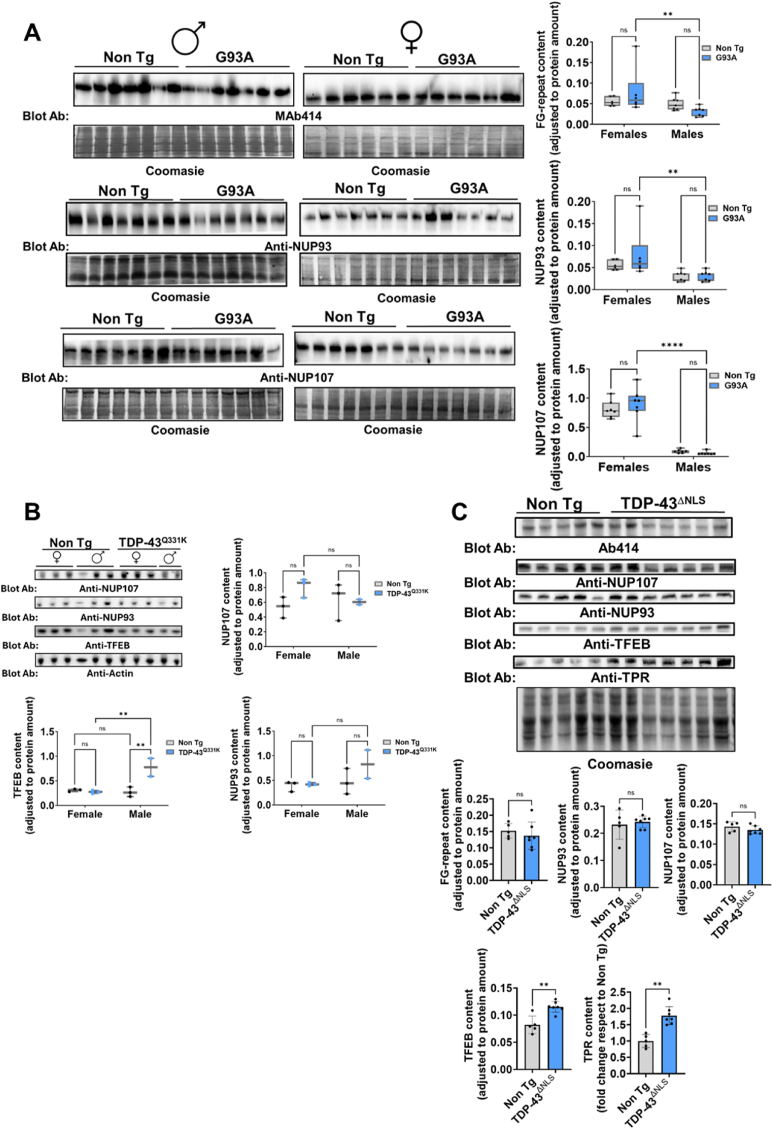
**SOD and TDP-43-based ALS preclinical models show altered nucleoporin content in spinal cord**. A) Representative western-blot images of total homogenates of lumbar spinal cords from female and male mice (90 days of age) from non-transgenic and G93A SOD mice, quantified in the right panel box-plots. B)Left panel, representative western-blot images of total homogenates of lumbar spinal cords from female and male mice of non-transgenic and TDP-43^Q331K^ transgenic mice, quantified in the right panel box-plot. C) Uper panel, representative western-blot images of total homogenates from lumbar spinal cord from male non-transgenic and ΔNLS-TDP-43 transgenic mice, quantified in the low panels. Box plots show mean values ± min to max values, with bars showing mean values ± SEM from n = 5–7 different mice, with ∗∗, and ∗∗∗∗ indicate p < 0.01 and p < 0.0001 significant differences between non-transgenic and transgenic mice or between male and female mice by Student's T test, Mann Whitney *U* test or post-hoc LSD after two-way ANOVA. ns: non statistically significant differences.

To confirm these results in other models of motor-neuron demise, we studied SOD-G93A and *TARDBP* transgenic models. In the G93A model, the lumbar spinal cord levels of Mab414-targeted NPC, Nup93, and Nup107 were affected in a sex and age-dependent manner by G93A overexpression. Thus, values of NPC were lower in male mice compared to female mice (Fig. 3A). Indeed, the content of FG-repeats containing NPC depended on the interaction between transgenesis and sex (p = 0.07, 11 % of total variance), but not Nup107 or Nup93, mainly dependent on sex at this age (100 days). At more advanced stages, G93A related differences were less evident (Supplemental Fig. 1). To ascertain if these changes were present in an experimental setup related to sporadic ALS, we employed two different *TARDBP*-related transgenic mice. In mice overexpressing the *TARDB* with the Q331K mutation, we found that the amount of Nup93 and Nup107 did not differ between sex and transgenic mice (Fig. 3B). Indeed, we found that transgenic male mice showed increased TFEB protein, potentially compatible with increased protein repair (Fig. 3B). As these TDP-43 mice did nothow a marked motor phenotype nor TDP-43 aggregates (data not shown), we evaluated NPC in the aggregation-prone ΔNLS-TDP-43. The results (Fig. 3C) indicate that Nup107, Nup93, and FG-repeats are not depleted in spinal cords from these models, albeit, as in the case of Q331K, the Tfeb amount was significantly increased (Fig. 3C). All in all, SOD and TDP-43-based ALS preclinical models do not generally alter nucleoporin content in whole lysates of the spinal cord, albeit they show increased Tfeb that may contribute to lysosome-based autophagic compensations. Interestingly, sex affects NPC levels in the spinal cord in the G93A mice. Notably, the three ALS models analyzed exhibit divergent NPC phenotypes: while G93A mice showed sex-dependent reductions in nucleoporin content, TDP-43Q331K and ΔNLS-TDP-43 mice presented modest or no consistent changes.

### 3.3 *NUP107* silencing alters autophagy and induces TDP-43 pathological characteristics *in vitro*

**Fig. 4:**
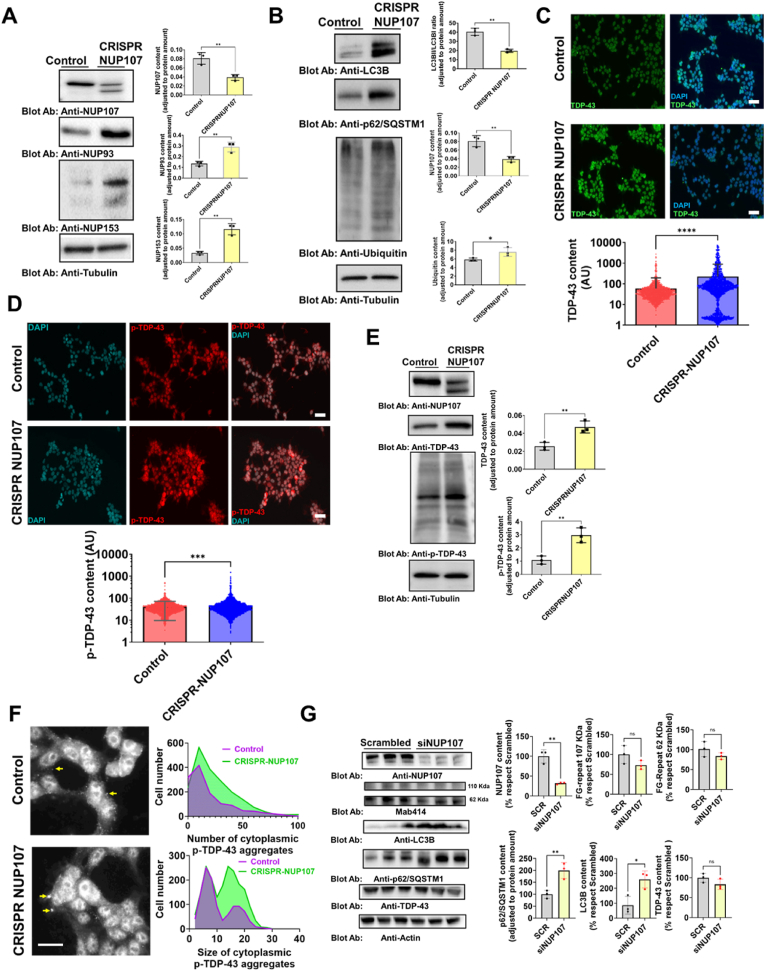
**NUP107 silencing alters autophagy and induces TDP-43 pathology in human cells**. A) Left panel indicates representative western-blot of different NUPs in CRISPR-mediated NUP-107 silencing of HEK293 cells. Right panel shows densitometry analyses of different experiments. B) Left panel shows representative western-blot of different autophagy and protein-turnover components in the same cells, with right panel indicating densitometric analyses. C) Confocal microscopy of TDP-43 immunoreactivity of HEK293 CRISPR-mediated NUP107 cells, showing altered TDP-43 immunoreactivity, quantified in the lower panel.D) Confocal microscopy of phospho-TDP-43 immunoreactivity of CRISPR-mediated NUP107-silenced HEK293 cells, showing altered TDP-43 immunoreactivity, quantified in lower panel microscopy. E) Western-blot analyses of TDP-43 and phospho-TDP-43 in CRISPR-mediated NUP107-silenced HEK293 cells, quantified in the right panel. F) Higher magnification of confocal microscopy images showing cytosolic aggregates of phospho-TDP-43 (yellow arrows) induced by CRISPR-mediated NUP107-silencing in HEK293 cells, quantified in the left panel (both size and number of phospho-TDP-43 aggregates). G)Left panel, representative western-blot analyses of NUPs and autophagy components in siRNA-mediated silencing of NUP107 in HeLa cells.Right panel shows the quantitative analyses. Bars show mean values ± SEM from N = 3–5 different experiments. ∗,∗∗, ∗∗∗ and ∗∗∗∗ indicate, respectively, p < 0.05, p < 0.01, p < 0.001 and p < 0.0001 significant differences between silenced or non silenced cells by Student's T test or Mann Whitney *U* test. In B and D, white scale bar lenght is 50 μm long and 30 μm in F. ns: non statistically significant differences.

Because NUPs play an important role in nucleo-cytoplasmatic transport, we decided to evaluate whether these proteins may be relevant in the TDP-43 delocalization as the main pathological characteristic of the disease. In this sense, *NUP107* and *NUP93* were silenced via a CRISPR system in HEK-293 cells and TDP-43 was analyzed. We did not succeed in getting stable clone cells with *NUP93* silenced (data not shown). The results demonstrate that partial depletion of NUP107 (Fig. 4A) was associated with an increased amount of other NPC, such as NUP153 and NUP93. Indeed, decreased NUP107 was accompanied by molecular traits compatible with increased autophagy flux, such as increased p62/SQSTM1 and LC3B amount, with increased ubiquitin staining (Fig. 4B). NUP107 silencing resulted in augmented levels of total TDP-43 and pTDP-43 analyzed by confocal microscopy (Fig. 4C and D) and by Western blot (Fig. 4E). Likewise, immunofluorescence analysis showed that the downregulation of NUP107 provoked a significant increase in the cytoplasmatic and nuclear phospho-TDP-43 aggregates in depleted *NUP107* cells in comparison to control cells (Fig. 4F). Similarly, the analysis of the aggregate size shows that these are significantly larger (Fig. 4F) in cells lacking NUP107 compared to control cells. We set up a transient *NUP107* silencing system based on siRNA in HeLa cells to validate these results independently. The results (Fig. 4G) demonstrate that in this case, albeit not finding significantly increased TDP-43 levels, similar autophagy changes (i.e. increased LC3B and p62/SQSTM1) were noted by silencing *NUP107*. Indeed, in these conditions, we evaluated TDP-43 splicing functions by measuring the potential buildup of cryptic exons in selected genes [35]. In line with a dose-dependent effect, transient NUP107 silencing effects did not induce the accumulation of cryptic exons in *PFKP*, *ATG4B*, or *GPSM2* (Supplemental Fig. 2). Nonetheless, transient *NUP107* silencing resulted in cell-cycle-dependent phenotypes of subcellular TDP-43 and NPC distribution (Supplemental Fig. 3). These findings align with prior work showing cell stress-induced cell cycle arrest and autophagy modulation via oxidative signaling [36], supporting the sensitivity of the G2/M phase to cell stress. Globally, these results illustrate that cells react to *NUP107* silencing, potentially altering autophagy and NPC architecture and inducing TDP-43 pathology in human cells.

### 3.4 Loss of TDP-43 induce alterations in nucleoporin homeostasis

**Fig. 5:**
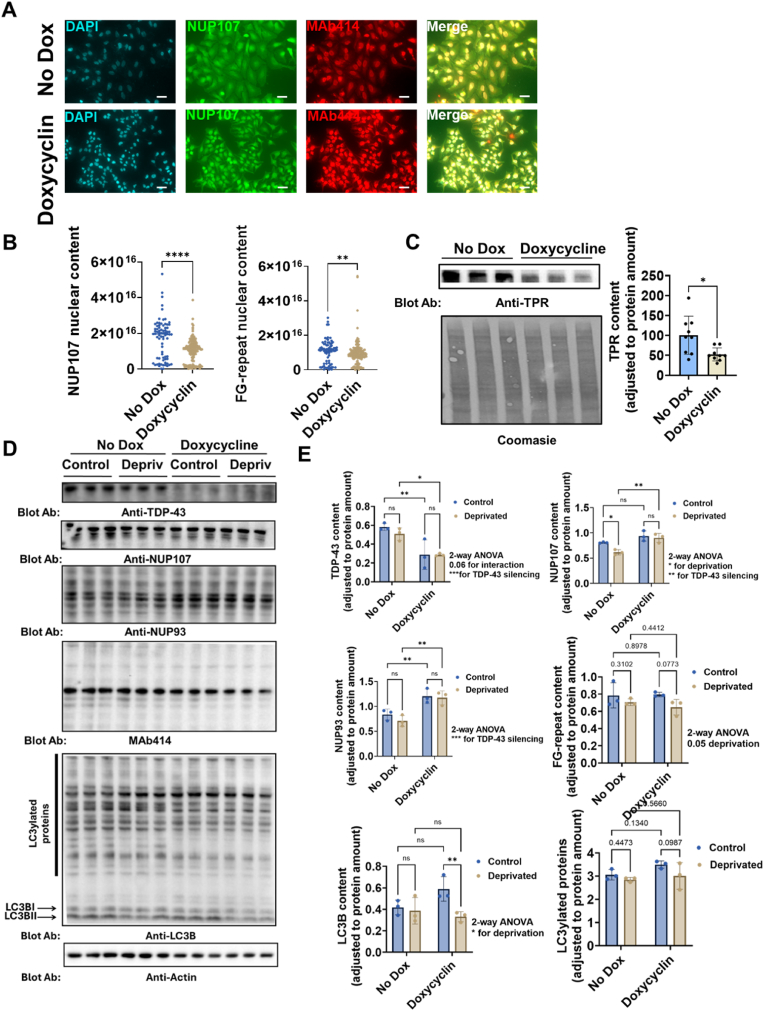
**Loss of TDP-43 induces alteration in nucleoporin homeostasis.** A) Representative immunofluorescence analyses showing NUP107 and MAb414 in TDP-43 silenced cells for a HeLa inducible silencing clone. B) Violin plot showing the quantitative analyses of nuclear (i.e. colocalizing with DAPI) content of NUP107 and MAb414 of cells in A. C) Left panel, western-blot analyses of TPR-content in HeLa cells with inducible TDP-43 silencing, with right panel showing quantitative analyses. D) Western-blot analyses of selected NUPs and LC3B in HeLA cells with doxycycline-inducible TDP-43 silencing under autophagy induction by 24 h of nutrient deprivation (Depriv). E) Quantitative analyses of western-blots shown in D. Bars show mean values ± SEM from N = 3–5 different experiments. ∗,∗∗, and ∗∗∗∗ indicate, respectively, p < 0.05, p < 0.01, and p < 0.0001 significant differences between silenced or non silenced cells by Student's T test, Mann Whitney *U* test or post-hoc LSD after two-way ANOVA. In A white scale bar lenght is 50 μm long. ns: non statistically significant differences.

As we evidenced that decreased NUP107 induced accumulation of TDP-43 in some conditions, we further explored whether there is a functional dependency between those two factors. To this end, we leveraged the recent development of a dox-inducible silencing of TDP-43 in the HeLa context [37]. Thus, in the face of decreasing TDP-43 levels, cells decrease the incorporation of NUP107 and FG-repeats NPC in nuclear locations (Fig. 5A and B). This is associated with decreased levels of the scaffold protein TPR required for the assembly of the NPC (Fig. 5C). Based on the above-observed activation of autophagy induced by loss of NUP107, we explored if autophagy activation (by nutrient deprivation) impacted the potential effects of TDP-43 loss in NPC homeostasis (Fig. 5D). The results show that TDP-43 loss increases total amounts of NUP107 and NUP93 without major changes in LC3ylation [38](Fig. 5E). Of note, TDP-43 loss increased cells sensitivity to importazol, an inhibitor of nuclear import, without affecting toxicity induced by nuclear export inhibition (Supplemental Fig. 4). Globally, these results are compatible with TDP-43 being relevant in the assembly of NPC via TPR, with cells reacting to potential loss of functional NPC, increasing expression of NUP107 and NUP93.

### 3.5 TDP-43 aggregating cell stressors impairs nucleoporin assembly with oxidative stress potentially modyfing FG-repeat containing NUPs *in vitro*

**Fig. 6:**
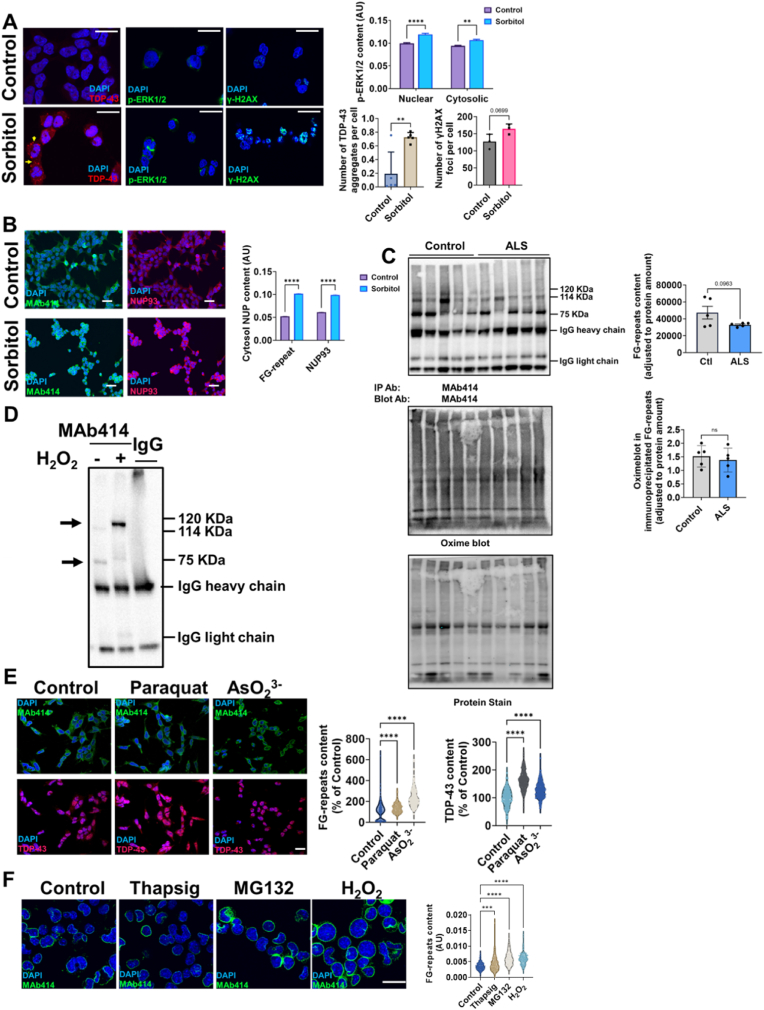
**TDP-43 aggregating cell stressors impairs nucleoporin assembly with oxidative stress potentially modyfing FG-repeat containing NUPs *in vitro***. A) Left panel, representative confocal imaging of TDP-43, *p*-ERK1/2 and γ-H2AX foci in HEK293 cells after osmotic stress (Sorbitol), indicating extranuclear aggregates (yellow arrow). Right panel shows quantitative analyses of these traits. B) Left panel representative confocal imaging showing cytosolic location of FG-repeats nucleoporin (MAb414) and NUP93 in HEK293 cells after osmotic stress, with right panel showing quantitative analyses of these characteristics. C) Left panel, representative western-blot of MAb414 immunoprecipitated from spinal cord samples from ALS patients and healthy, age and sex-matched individuals, showing potentially oxidative modifications by oxime blot analyses. Right panel shows the quantitative analyses after densitometry. D) *In vitro* oxidative modification of FG-repeats containing NUPs as shown by anti-DNPH western-blot analyses. Arrows show the change of electrophoretic mobility, compatible with oxidative modification. E) Left panel shows confocal imaging of FG-repeat NUPs (MAb414) and TDP-43 distribution in SH-SY5Y cells after oxidative treatments, with right panel showing quantitative analyses. F) Left panel confocal imaging of TDP-43 aggregating cell stressors (ER stress, proteasome stress and oxidative stress) affecting FG-repeat subcellular distribution in Neuro2A cells, as quantified in right panel violin plots. Bars show mean values ± SEM from N = 4–6 different experiments (except for D, 3 experiments). ∗∗,∗∗∗, and ∗∗∗∗ denote, respectively, p < 0.01, p < 0.001, and p < 0.0001 significant differences between stressed or non stressed cells by Student's T test, Mann Whitney *U* test or post-hoc LSD after one-way ANOVA. In A and F, white scale bar lenght are 30 μm long, while as in B and E are 50 μm. ns: non statistically significant differences.

We evaluated whether cell stressors inducing TDP-43 pathological traits also alter NPC's distribution in Neuro2a cells after osmotic stress. As shown in Fig. 6A, osmotic stress-induced aggregation in TDP-43 and *p*-ERK [39] is reminiscent of ALS pathology. TDP-43 aggregation was involved, as well as the cytosolic buildup of phospho-TDP-43 aggregates and loss of splicing functions (Supplemental Fig. 5). In these conditions, FG repeats and NUP93 cellular immunoreactivity increased (Fig. 6B), in line with the potential disruption of NPC assembly (Supplemental Fig. 3). As osmotic stress included oxidative stress (evidenced by increased γH2AX foci, Fig. 6A), and based on the long life of NPC [22,40], we evaluated if isolated NPC from the brain cortex showed oxidative modifications. We set up the recently developed oxime blot assay (Supplemental Fig. 6, showing that TDP-43 loss does not increase general protein carbonyl content) and employed it in NPC immunoprecipitates. The results (Fig. 6C) are compatible with potentially modified FG-repeats *in vivo*, though the modification degree seems unrelated to ALS. Independent *in vitro* studies show that FG-repeats from immunopurified NPC isolated from the brain cortex can be oxidatively modified by H2O2, increasing the degree of carbonyl content and changing its structure (Fig. 6D). These results were validated independently, as other cellular systems under several oxidative stressors, such as arsenite or paraquat, exhibited increased FG-repeat levels, potentially associated with decreased NPC assembly, in line with increased TDP-43 levels (Fig. 6E).Indeed, TDP-43 loss in the HeLa did not enhance toxicity of arsenite, as based on dose-dependence studies (Supplemental Fig. 7). As an orthogonal validation for oxidative modification of NUPs we evaluated previous data (employing mass-spectrometry) and *in silico* predictions. Thus, data on Carbonyldb show carbonylation of specific Pro residues in nucleoporin NSP1 after H2O2 stress [41], of His in NUP155 *in vivo* [42], and Lys residues in Nuclear pore glycoprotein p62 in HeLa cells [43]. *In silico* predictions of carbonylation (supplemental dataset 1) indicate that, curiosly, scaffold NUPs seem spared against carbonylation, while susceptible Pro, Arg, Lys are found in FG-repeat NUPs and membrane-associated NUPs as POM121, NUP98, NUP153 and NUP62, supporting our results. Of note, studies with other ALS-related cellular stressors (ER-stress and proteasome stress), previously involved in TDP-43 pathological traits [39], also induce increased cell levels of FG-repeat NUPs, suggesting impaired NPC assembly in line with decreased TDP-43. Thus, TDP-43 aggregating cell stressors impair nucleoporin assembly with oxidative stress, potentially modifying FG-repeat-containing NUPs *in vitro*.

## 4 Discussion

Our findings suggest NPC dysfunction, particularly involving its components NUP107 and FG-repeat NUPs, as a mechanistic contributor to TDP-43 pathology in ALS. We demonstrate that NUP107 loss is sufficient to induce TDP-43 mislocalization and aggregation, autophagy dysfunction, and altered NPC architecture in human cells, aligning with prior work linking NUP depletion to impaired nucleocytoplasmic transport and neuronal stress responses [2,17] Reciprocally, TDP-43 silencing disrupted NPC composition and reduced TPR expression, underscoring a bidirectional relationship between TDP-43 and NPC integrity [16,44]. Although we observed that TDP-43 knockdown perturbs NPC composition, we did not assess whether overexpression or restoration of TDP-43 can reverse these changes. Such complementary gain-of-function experiments would provide valuable insight into causal relationships.

In ALS post-mortem tissues and preclinical models, NPC alterations varied by sex and disease stage. In G93A mice, reduced NUP content was more evident in males, consistent with known sex differences in disease progression and estrogen-mediated neuroprotection[[45], [46], [47]]. Estradiol has been shown to modulate NPC number and assembly in mammalian neurons [48,49], possibly via Nrf1-dependent mitochondrial pathways [47]. Our data reinforce this sex-specific vulnerability and link it to nucleoporin integrity. Sex-based differences in nucleoporin levels—more pronounced in male G93A mice—may reflect the influence of estrogens on nuclear pore function and assembly. Estradiol has been shown to modulate NPC architecture, potentially via ERα-Nup133 signaling or mitochondrial Nrf1 activation, offering a possible explanation for neuroprotection in females[[45], [46], [47]]. All in all, discrepancies in the TDP-43 vs G93A mice may reflect differences in disease progression, protein aggregation, or compensatory mechanisms operating in the TDP-43 models. Of note, based on data of oxime blot and in arsenite dose-response measurements, TDP-43 loss in cell lines does not increase generally protein oxidative damage, demonstrating the resilience of remaining cells.

NUP107 emerged as a central node in this pathogenic axis in ALS. Its depletion led to accumulation of ubiquitinated proteins, increased LC3B and p62/SQSTM1, and formation of cytoplasmic and nuclear TDP-43 aggregates—phenocopying key ALS pathological features [39]. Conversely, TDP-43 knockdown triggered loss of NPC components, including TPR, and increased TFEB expression, a master regulator of lysosomal and mitochondrial stress responses [50,51] This suggests a compensatory activation of autophagy and quality control programs in response to NPC disruption and proteostasis imbalance [38,52]. Recent studies have shown that TDP-43 pathology and NPC failure are linked via ESCRT-III dysfunction, with CHMP7 accumulation and impaired nuclear envelope surveillance contributing to neuronal injury [3,53]. The observed TFEB upregulation in our models may reflect compensatory responses to lysosomal damage and impaired autophagic flux—potentially exacerbated by ESCRT-III deficits and ATG4B dysfunction previously described in ALS [38,54]. Nonetheless, while such TFEB upregulation, observed in several TDP-43 models could compensate these lysosomal and proteostasic stress, its functional contribution to NPC (dys)homeostasis in ALS remains to be fully demonstrated, which may be focus of future studies. However, the present interactions place NPC integrity at the intersection of proteostasis, mitochondrial health, and nucleocytoplasmic transport.

We further show that TDP-43 aggregating stressors, including oxidative and osmotic insults, induce mislocalization of FG-repeat NUPs and impair NPC assembly. These findings echo previous reports of stress-induced NPC injury and cytoplasmic accumulation of NUPs in neurodegenerative models [55,56]. Using oxime blot and DNPH methods, we found that FG-repeat NUPs are susceptible to oxidative modification *in vitro* and in brain tissue, supporting the idea that these long-lived proteins are redox-sensitive and may accumulate damage with age or stress [20,31,40]. Also, functional assays evaluating nuclear import/export dynamics will be critical to establish a causal link between oxidative modifications and NPC impairment. Although this study did not assess antioxidant rescue, future work should determine whether pharmacological reduction of oxidative stress can attenuate NPC disruption and TDP-43 mislocalization, potentially revealing therapeutic opportunities.

While our data support a mechanistic link between NPC disruption and ALS pathology, several limitations warrant consideration. First, although we used multiple complementary models, some findings remain correlative. This study primarily employed non-neuronal human and murine cell lines for mechanistic clarity. While informative, future validation in motor neuron-specific systems — particularly those derived from ALS patient iPSCs — will be critical to confirm the relevance of NPC alterations in motor neuron degeneration. Nonetheless, the marked abundance of NUP107 and TPR in motor neurons in sections of human spinal cord, suggest the importance of these proteins in these ALS-relevant cells. Second, bulk tissue analyses may obscure neuron-specific NPC alterations. Third, the absence of dose-dependent validation for oxidative and other cell stress treatments limits mechanistic interpretation and generalizability, which should be addressed in future work, refining our understanding of potential threshold effects and neurotoxicity. Fourth, while our data support a pathological role for NUP107 depletion, gain-of-function studies—such as NUP107 overexpression—would be necessary to confirm whether restoring its levels can mitigate TDP-43 pathology. Also, while our findings support oxidative modifications in FG-repeat nucleoporins, we acknowledge that oxime blotting and DNPH derivatization do not provide residue-specific resolution. Future studies using redox proteomics approaches, such as mass spectrometry, will be essential to precisely identify carbonylated residues and confirm their functional impact. Finally the functional impact of oxidative NUP modifications on transport dynamics remains to be fully elucidated. Despite these caveats, our work establishes NPC injury—particularly involving NUP107—as a critical and dynamic driver of TDP-43 mislocalization and cellular stress in ALS. These insights point toward novel therapeutic strategies aimed at restoring nucleocytoplasmic transport, supporting NPC assembly, and mitigating proteostatic overload in ALS and related neurodegenerative disorders.

## CRediT authorship contribution statement

**O. Ramírez-Núñez:** Visualization, Validation, Methodology, Investigation. **S. Rico-Ríos:** Resources, Methodology, Investigation. **P. Torres:** Validation, Supervision, Software. **V. Ayala:** Project administration, Formal analysis, Data curation. **A. Fernàndez-Bernal:** Methodology, Investigation, Conceptualization. **M. Ceron-Codorniu:** Visualization, Validation, Resources. **P. Andrés-Benito:** Writing – original draft, Visualization, Data curation. **A. Vinyals:** Methodology, Investigation. **S. Maqsood:** Methodology, Investigation. **I. Ferrer:** Writing – review & editing, Supervision, Resources. **R. Pamplona:** Project administration, Methodology, Funding acquisition. **M. Portero-Otin:** Writing – review & editing, Writing – original draft, Visualization, Supervision.

## Declaration of competing interest

The authors declare that they have no known competing financial interests or personal relationships that could have appeared to influence the work reported in this paper.

## Data Availability

Data will be made available on request.
