# Supplementary material for: Nuclear pore complex dysfunction drives TDP-43 pathology in ALS
Source: Redox Biol. 2025 Aug 14;86:103824. doi: 10.1016/j.redox.2025.103824 (PMC12390953; doi:10.1016/j.redox.2025.103824)
Supplement: Multimedia component 2 [file mmc2.docx]

**Nuclear Pore Complex Dysfunction Drives TDP-43 Pathology in ALS**

**Supplemental Table 1.**

**Reagents/Tools Table**

| **Reagent/Resource** | **Reference or Source** | **Identifier or Catalog Number** |
| --- | --- | --- |
| **Experimental Models** |  |  |
| Patient samples | Institute of Neuropathology (Universitat de Barcelona) | This study. Table 1 |
| B6.Cg-Tg(SOD1-^G93A^)1Gur/J transgenic mice (G93A) | The Jackson Laboratory | Stock No. 002726 |
| B6.Cg-Tg(Prnp-*TARDBP*^Q331K^) transgenic mice (Q331K) | The Jackson Laboratory | Stock No. 017933 |
| B6;C3-Tg(NEFH-tTA)8Vle Tg(tetO-TARDBP)12Vle/J (NLS-TDP) | The Jackson Laboratory | Stock No. 028413 |
| Non-transgenic littermates | Non-transgenic mice after genotyping according to The Jackson Laboratory |  |
| Neuro-2A (mouse neuroblastoma) | ATCC | CCL-131 |
| SH-SY5Y (human neuroblastoma) | ATCC | CRL-2266 |
| HeLa (human cervical carcinoma) | ATCC | CCL-2 |
| PLKO (HeLa clone with tetracycline-inducible silencing of *TARDBP*) | Ceron-Codorniu et al., 2024 |  |
| HEK-293 (human embryonic kidney) | ATCC | CRL-1573 |
| IPSc | Coriell | Cat.#ND35663,Cat.#ND41866 |
| **Recombinant DNA** |  |  |
| CRISPR/Cas9 plasmids for NUP107 knockdown | Santa Cruz Biotechnology | sc-405252-KO-2 |
| CRISPR/Cas9 plasmids for NUP93 knockdown | Santa Cruz Biotechnology | sc-402116-HDR |
| siRNA for NUP107 | Sigma | NM_020401 |
| Tet-pLKO-puro | Addgene | Plasmid #21915 |
| psPAX2 | Addgene | Plasmid #12260 |
| pMD2.G | Addgene | Plasmid #12259 |
| **Antibodies (dilution for WB; for ICC)** |  |  |
| MAb414 (1:500; 1:200 ) | Abcam | ab24609 |
| Anti-NUP107 (1:1000; 1:200) | Abcam | ab73290 |
| Anti-NUP93 (1:1000;1:200) | Abcam | ab92776 |
| Anti-DNPH (1:10000) | Sigma | D9656 |
| Anti-NUP153 (1:1000) | Abcam | ab81563 |
| TDP-43 (WB 1:1000;ICC 1:250) | Proteintech | 10782-2-AP |
| Anti–phospho-TDP-43 pSer 409/410 (1:1000) | Cosmo | --CAC-TIPPTDM01 |
| Anti–p62/SQSTM1 (1:1000) | Cell Signaling Technologies | 5114 |
| Anti–LC3B (1:2000) | Cell Signaling Technologies | 2775 |
| Anti- γ H2AX (ICC 1:200) | Abcam | ab2893 |
| Anti-pERK1/2 (1:200) | Upstate Biotechnology | 05-481 |
| Anti-Ubiquitin (1:100) | Sigma | U5379 |
| Anti-Tubulin β III (ICC 1:200) | Millipore | MAB1637 |
| Anti-Tubulin (1:5000) | Abcam | ab7291 |
| Anti-TFEB (1:1000) | Sigma | SAB4503154 |
| Anti-TPR (1:1000) | Sigma | HPA019661 |
| Anti–β-actin | sigma | a5441 |
| Anti-mouse IgG, HRP conjugated (1:30000) | Amersham | NA931V |
| Anti-rabbit IgG, HRP conjugated (1:50000) | Pierce | 31460 |
| Anti-rabbit IgG,  Alexa-Fluor® 546  Conjugate (ICC: 1:500) | Thermo Fisher Scientific | A11010 |
| Anti-mouse IgG,  Alexa-Fluor® 546  Conjugate (ICC: 1:500) | Invitrogen | A11060 |
| Anti-mouse IgG,  Alexa-Fluor® 488  Conjugate (ICC: 1:500) | Thermo Fisher Scientific | A28175 |
| Anti-rabbit IgG,  Alexa-Fluor® 488  Conjugate (ICC: 1:500) | Thermo Fisher Scientific | A11008 |
| R-Phycoerythrin (RPE) conjugation kit | Abcam | ab102918 |
| Allophycocyanin (APC) conjugation kit | Abcam | ab201807 |
| APC/Cy7 conjugation kit | Abcam | ab102859 |
| **Oligonucleotides and other sequence-based reagents** |  |  |
| shRNA targeting *TARDBP* | Millipore-Sigma | TRCN0000016038 |
| shRNA targeting *NUP107* | Millipore-Sigma | Not stated (GCUGCAAAAGAAGUAUUUG) |
| Primers for RT-qPCR | Millipore-Sigma | Supplemental Table 2 |
| shRNA scrambled | Millipore-Sigma | Not stated (UAAGGCUAUGAAGAGAUAC) |
| **Chemicals, Enzymes and other reagents** |  |  |
| DMEM (Dulbecco’s Modified Eagle Medium) | Thermo Fisher Scientific | 10564011 |
| DMEM/F12 | Thermo Fisher Scientific | 11320033 |
| HBSS | Gibco | 14025-050 |
| FBS (Fetal Bovine Serum) | Thermo Fisher Scientific | A5256701 |
| Plasmocin® prophylactic | Invivogen | ant-mpp |
| TRI Reagent | **Thermo Fisher Scientific** | **AM9738** |
| Penicillin–Streptomycin (P/S) | Thermo Fisher Scientific | 15070063 |
| **TaqMan Reverse Transcription Reagent** | **Thermo Fisher Scientific** | **N8080234** |
| **SYBR Select Master Mix for CFX** | **Thermo Fisher Scientific** | **4472937** |
| Puromycin | Sigma | P9620 |
| Thapsigargin | Sigma | T9033 |
| Sorbitol | Sigma | S1876 |
| Menadione | Sigma | M5625 |
| Paraquat | Sigma | 856177 |
| Lipofectamine® 3000 | Thermo Fisher Scientific | L3000001 |
| H₂O₂ | Sigma | 31642-M |
| MG132 | Sigma | C2211 |
| Leptomycin B | Sigma | L2913 |
| Importazol | Sigma | SML0341 |
| RIPA buffer (50 mM Tris-HCl (pH 8.0), 150 mM NaCl,  1% Triton X-100, 0.5% Sodium deoxycholate, 0.1% SDS and 1 mM EDTA) | Sigma |  |
| TKM buffer (50 mM Tris (pH 8.0), 25 mM KCl, 5 mM MgCl_2)_ | Sigma |  |
| Normal Goat Serum | Thermo Fisher Scientific | 31872 |
| Normal Horse Serum | Thermo Fisher Scientific | 31874 |
| Halt Protease and Phosphatase inhibitor cocktail | Thermo Fisher Scientific | 78440 |
| Sucrose (for nuclear isolation) | Sigma | S0389 |
| Paraformaldehyde (PFA) | Sigma | 47608 |
| DAPI | Sigma | MBD0015 |
| Poly-L-lysine | Sigma | P4707 |
| Bradford Assay reagents | Bio-Rad | **5000001** |
| No-Stain™ Protein Labeling Reagent | Thermo Fisher Scientific | A44449 |
| **I-Block™ Protein-Based Blocking Reagent** | Thermo Fisher Scientific | T2015 |
| **SurePAGE™ Precast gels** | Genscript | M00654 |
| **Immobilon™ ECL Ultra Western HRP Substrate** | **Merck Millipore** | **WBKLS0500** |
| **Immobilon™ Western HRP Substrate (for oxime blot)** | **Merck Millipore** | **WBKLS0050** |
| **Immobilon-P™ PVDF membrane** | **Merck Millipore** | **#IPVH00010** |
| **Coomassie Brilliant Blue G** | **Sigma** | **27815** |
| **EZ-Link™ Alkoxyamine-PEG4-Biotin** | Thermo Fisher Scientific | **26137** |
| **p-Phenylenediamine** | **Sigma** | **P6001** |
| **Hydroxylamine (50%)** | **Sigma** | **8144410100** |
| **Dithiothreitol (DTT)** | **Sigma** | **D0632-5G** |
| **Streptavidin-horseradish peroxidase conjugate (1:2000 in 10 mL)** | **Cytiva** | **RPN1231V** |
| **Oxime blot RIPA lysis buffer (**10 mM sodium phosphate - pH 7.5, 150 mM NaCl, 1% NP-40, and 0.1% sodium deoxycholate, with phosphatase and protease inhibitors added) | **Sigma** |  |
| **10 mM 2,4-dinitrophenylhydrazine(DNPH) solution (dissolved in 10% trifluoroacetic acid in H_2_O)** | Sigma | **D-2630** |
| **RS solution (**170 mL Tris 2M 30% glycerol + 30 mL B-Mercaptoetanol) | Sigma |  |
| **Dynabeads protein G** | Invitrogen | **10004D** |
| **Software** |  |  |
| GraphPad Prism | GraphPad Software | Version 10.4.1 |
| ImageJ | NIH | <https://imagej.nih.gov/ij/> |
| **ImageLab** | **Bio-Rad** | Version 6.1 |
| FlowJo or BD CellQuest (flow cytometry) | BD Biosciences | Not stated |
| Zeiss LSM confocal software | Carl Zeiss | Not stated |
| **Other** |  |  |
| BD FACSCalibur™ Flow Cytometer | BD Biosciences | Not stated |
| Zeiss LSM Confocal Microscope (LSM series) | Carl Zeiss | Not stated |
| **Ultra-Turrax homogenizer** | IKA | **L_IKA_3001** |

**Supplemental Table 2. Primers employed for qRT-PCR analyses.**

| **Genes** | **Forward** | **Reverse** |
| --- | --- | --- |
| TOTAL *ATG4B* | 5′-AACGCATTCATCGACAGGAAG-3′ | 5′-TTTGCGCTATCTGGTGAATGG-3′ |
| CRYPTIC *ATG4B* | 5′-CTGAGTGTGCATGGATGAGTG-3′ | 5′-TTGCTGGCACCAATCATTGAA-3′ |
| *TARDBP* | 5′-CTGCGGGAGTTCTTCTCTCA-3’ | 5′-CGCAATCTGATCATCTGCAA-3’ |
| TOTAL *GPSM2* | 5’-GGACGTGCCTTTGGAAATCTT-3′ | 5′-TTTGCAATAAGGAGACGCTGC-3’ |
| CRYPTIC *GPSM2* | 5′-GTGTGTATGAGAGAGAGAGCGA-3′ | 5′-AGAAGCTTCCATTCTGTTCATCA-3′ |
| TOTAL *PFKP* | 5′-GACCTTCGTTCTGGAGGTGAT-3′ | 5′-CACGGTTCTCCGAGAGTTTG-3′ |
| CRYPTIC *PFKP* | 5′-ACGTTTGCAAAACATCAGGAG-3′ | 5′-GCCTTCAACTCTCCGTTCAC-3′ |
| *GAPDH* | 5′-CCCTTCATTGACCTCAACTACATG-3′ | 5′-TGGGATTTCCATTGATGACAAG-3′ |

**SUPPLEMENTAL FIGURES**

**
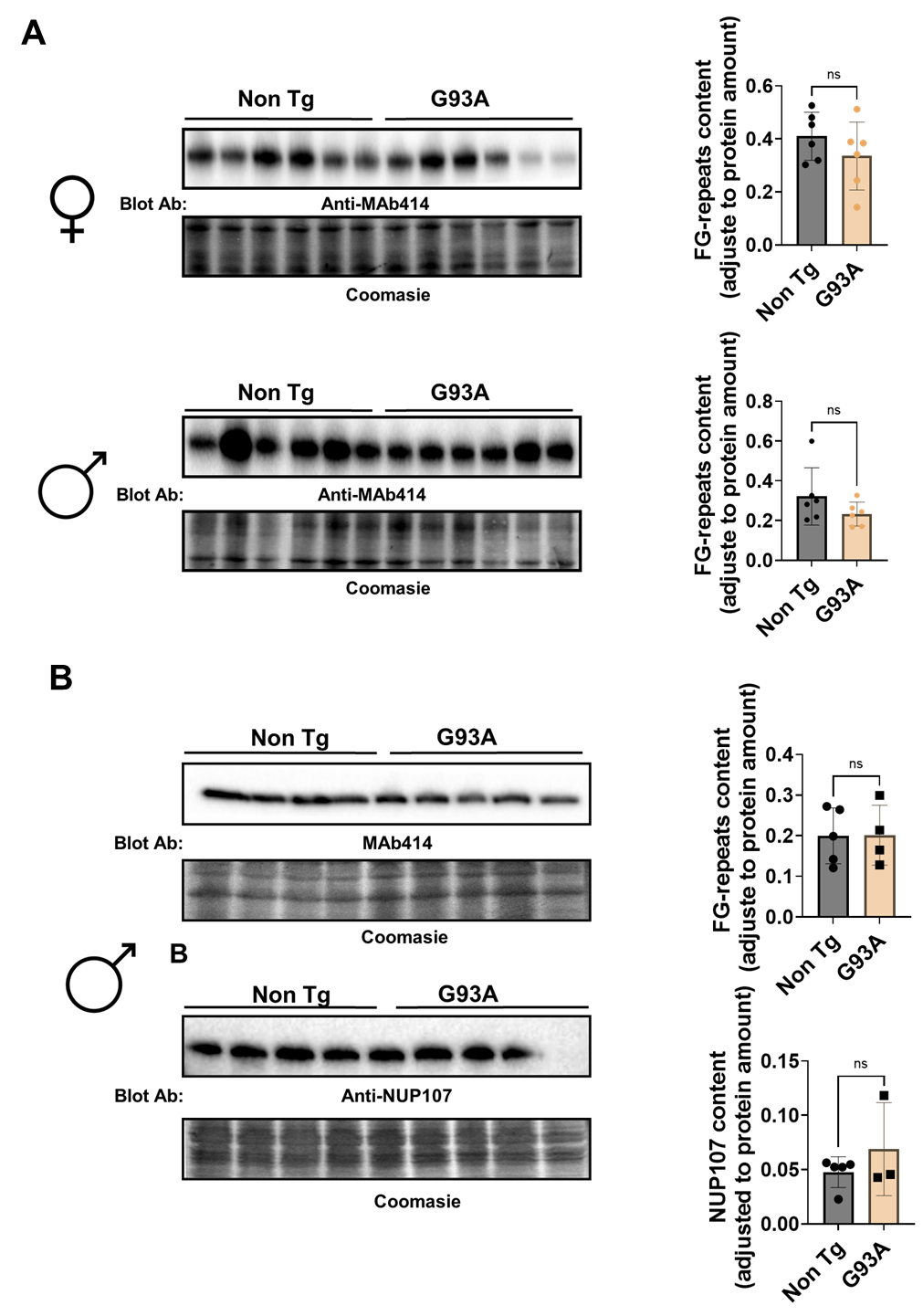
**

Supplemental Figure 1.**SOD G93A do not show altered nucleoporin content in spinal cord irrespectively of age.** A) Representative western-blot images of total homogenates of lumbar spinal cords from female and male mice (60days of age) from non-transgenic and G93A SOD mice, quantified in the right panel graphs. B)Left panel, representative western-blot images of total homogenates of lumbar spinal cords from male at end stage, quantified in the right panel graphs. Bars show mean values ± SEM from n=5-7 different mice, with non significant differences found in Student’s T test.


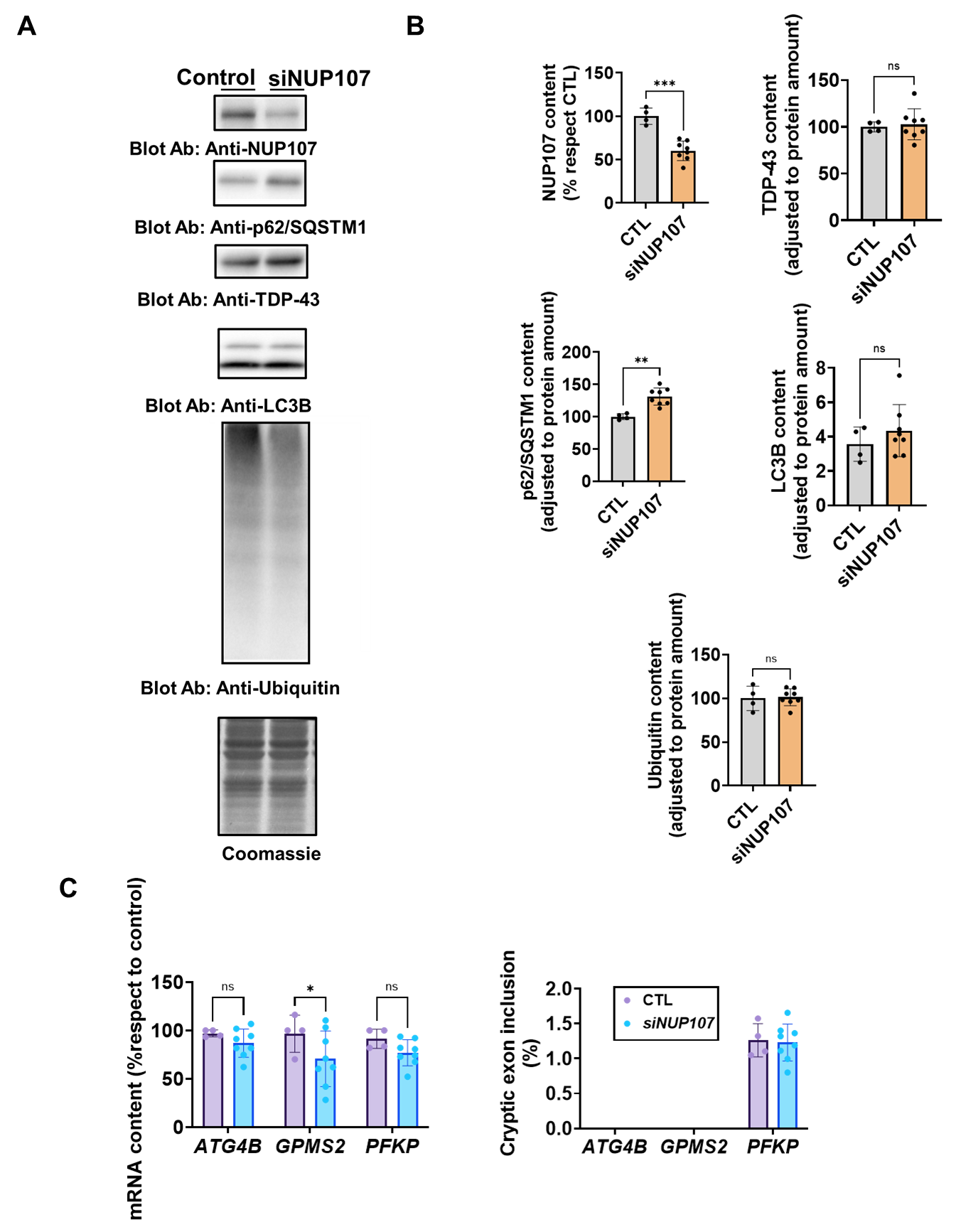


Supplemental Figure 2. **Transient NUP107 silencing alters autophagy in human cells** . A) Left panel indicates representative western-blot of different NUPs and different autophagy and protein-turnover components in the same cells in siRNA-mediated NUP-107 silencing of HeLa cells, B) Densitometric analyses of western-blots in A, and C) RT-qPCR of mRNAs (left) and cryptic exon inclusion (right, not detected for all genes) of the same cells in *ATG4B, GPSM2* and *PFKP*. Bars show mean values ± SEM from N=3 different experiments with two different siRNA sequences . *,**, and *** denote, respectively, p<0.05, p<0.01, and p<0.001 significant differences between silenced or non silenced cells by Student’s T test,Mann Whitney U test or post-hoc LSD after two-way ANOVA. ns: non statistically significant differences.


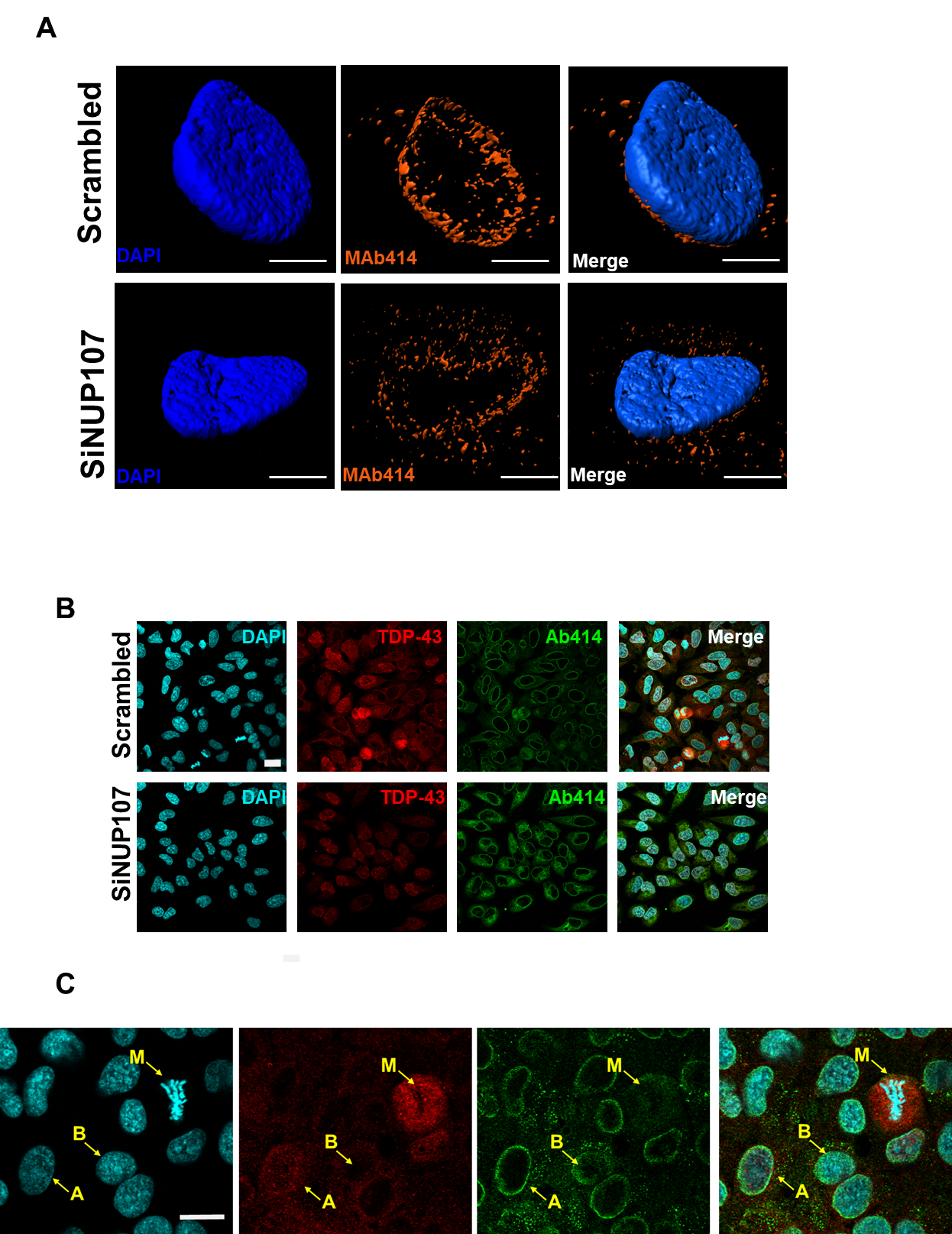


SupplementaL Figure 3. **Transient NUP107 silencing alters NPC assembly and effects on TDP-43 pathology depend on cell cycle.** A) High magnification of confocal microscopy based 3D reconstruction of nuclear pore complexes (MAb414 staining) in a single nuclei showing loss of physiological assembly after NUP107 silencing in HeLa cells. B)Confocal microscopy imaging showing the effect of NUP107 silencing on TDP-43 and Ab414 distribution. As illustrated by high magnification images in C), silencing of NUP107 diminished the number of cells in metaphase (M), whose TDP-43 cellular content is higher than quiescent cells. Two phenotypes of nucleoporin staining appear, one consisting with complete nuclear envelope (A type) and one with disordered nuclear envelope (B type). The nuclear distribution of TDP-43 is dependent on these phenotypes, with B type cells showing lower nuclear amount of TDP-43 than A type cells. In A white scale bar lenght are 5 μm long, while as in B and C are 10 μm.


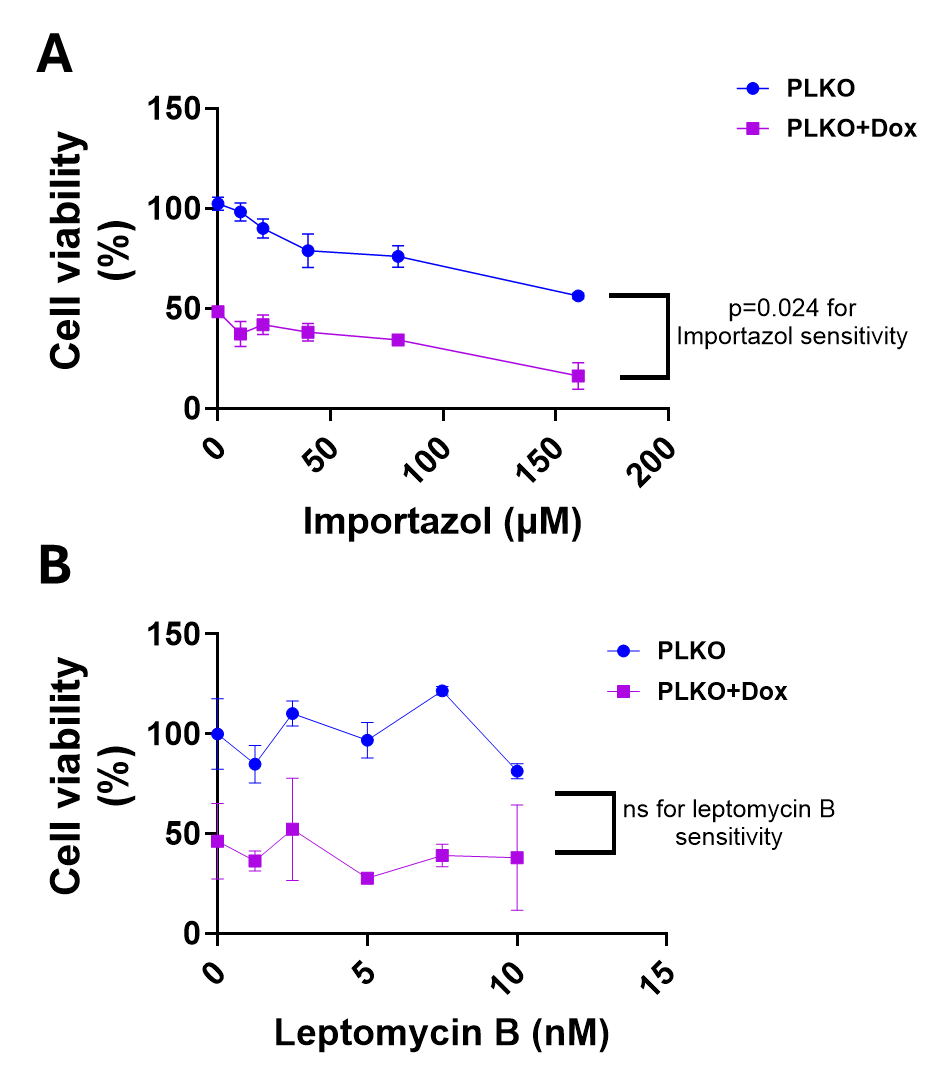


SupplementaL Figure 4. **Loss of TDP-43 sensitizes human cells to blockage of importin-β-mediated nuclear import.** Cell viability of a HeLa clone (designed PLKO) is lowered by *TARDBP*  silencing under doxycycline control (DOX). Cells were exposed to a dose-response disruption of NPC by either inhibition of importin-β-mediated transport by incubation with importazol (60 min exposure) (A), or inhibition of nuclear export mediated by the karyopherin protein chromosomal region maintenance 1 (CRM1), by incubation with leptomycin B (30 min exposure) (B). In A) slopes of linear relationships between dose and cell viability were significantly different in vehicle treated or *TARDBP* silenced cells (PLKO slope -0,3421 to -0,2002 (95%CI); PLKO+Dox slope -0,2289 to -0,1127; F=5.92, p=0.024 for equivalence between slopes), while as for B) slopes did not differed ( PLKO slope -3,242 to 2,923; PLKO+Dox slope -3,646 to 2,005; F= 0.12, p=0.72 for equivalence between slopes).


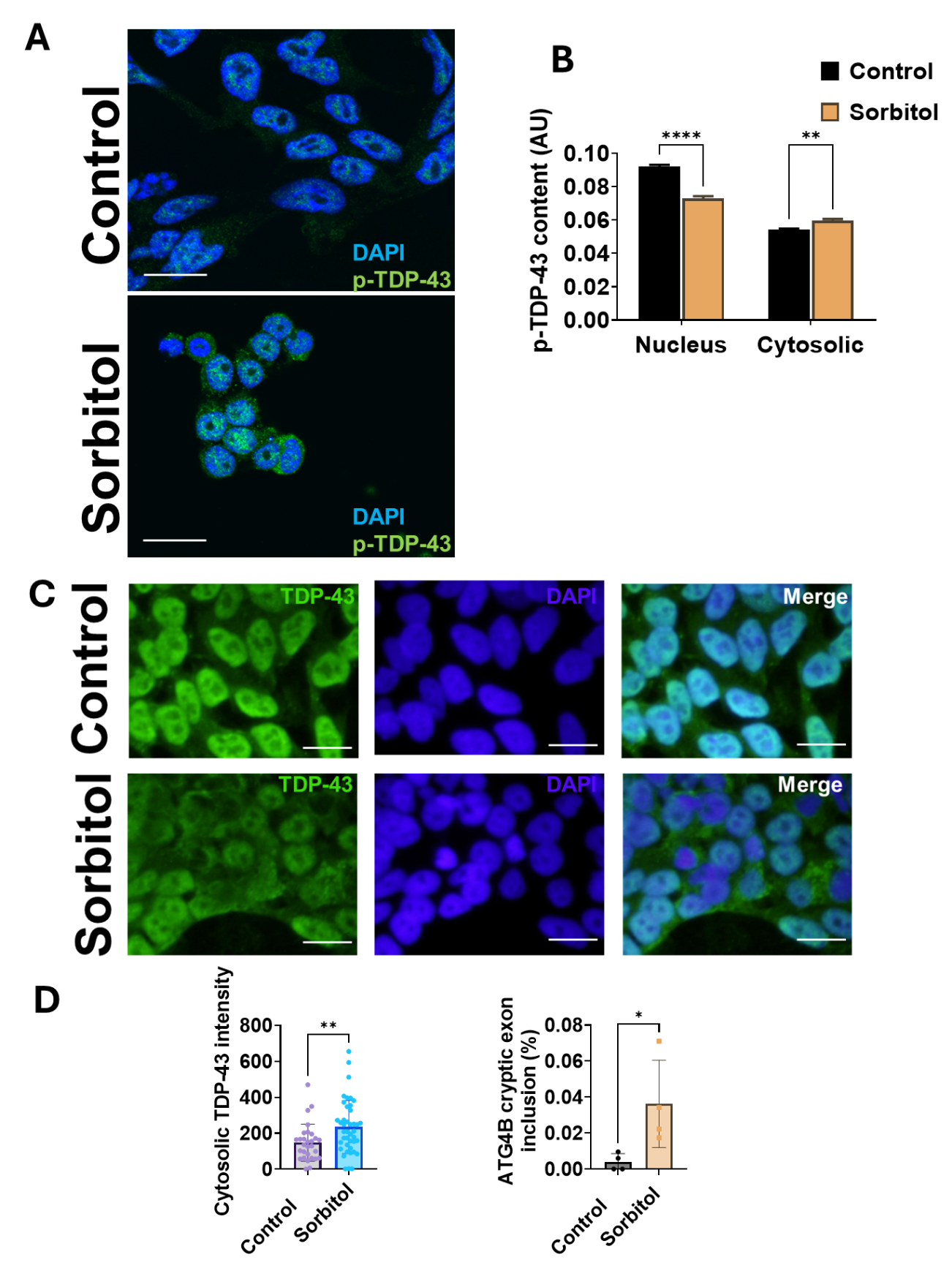


Supplemental Figure 5. **Osmotic stress induces TDP-43 pathology with loss of function.** A) Left panel, representative confocal imaging of phospho-TDP-43 cytosolic aggregates in HEK293 cells after osmotic stress (Sorbitol) B) Quantitative analyses of these traits, compatible with disturbed phospho-TDP-43 nucleocytosolic transport. C) Representative confocal imaging showing cytosolic location of TDP-43 aggregates in IPSc after osmotic stress, D) RT-qPCR of mRNAs (left) and cryptic exon inclusion (right) of the same cells in *ATG4B*. Bars show mean values ± SEM from N=3 different experiments. **, and ****denote, respectively, p<0.01, and p<0.0001 significant differences between stressed or non stressed cells by post-hoc LSD after two-way ANOVA.


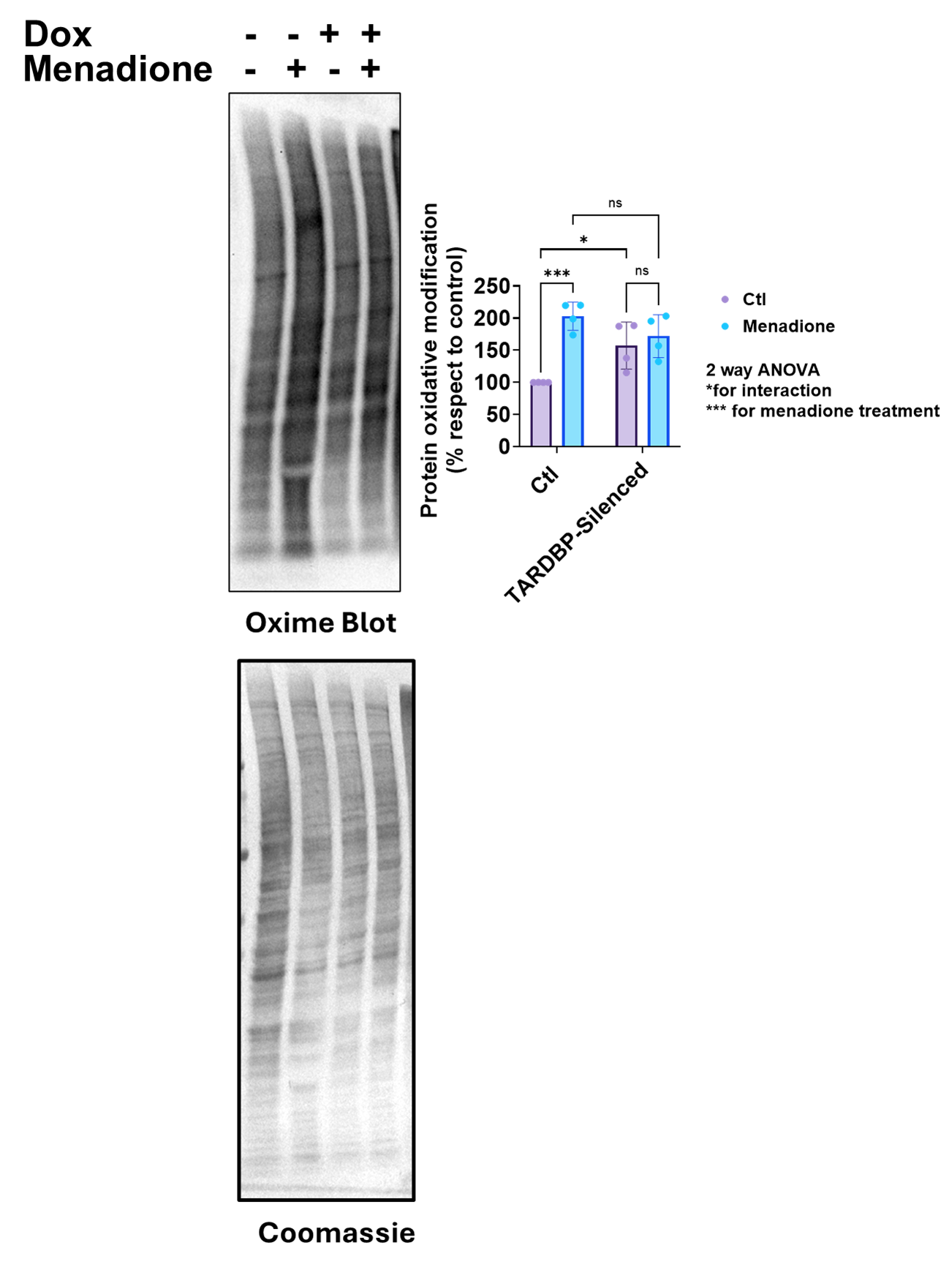


Supplemental Figure 6. **Oxime blot validation in cell culture**. Left panel indicates representative oxime blot and effects of treatment by menadione and TDP-43 silencing in a dox-inducible clone of TDP-43 silencing in HeLa. Right panel shows quantitative analyses of western-blots. Bars show mean values ± SEM from N=3 different experiments. *, and *** denote, respectively, p<0.05, and p<0.001 significant differences between conditions by post-hoc LSD after two-way ANOVA. ns: non statistically significant differences.

Supplemental Figure 7. **Dose dependent arsenite toxicity is independent on TDP-43 expresion in HeLa PLKO clone**. PLKO cells were treated with vehicle (PLKO) or its *TARDBP* mRNA expression silenced by doxycycline (DOX, 72 h), and exposed (3h) to sodium arsenite at different dosis. As indicated by the post-hoc LSD analyses after two-way ANOVA non statiscally significant differences were found for Dox treatment (2.25% of total variance explained, p=0.369), while as arsenite concentration influenced significantly cell viability (61% of total variance explained, p=0.012), and interaction between both factors being not significant (5% of total variance explained, p=0.84)
